# Supplementary material for: Effectiveness and user experiences of a valgus brace in patients with knee osteoarthritis: A mixed-method randomised controlled trial
Source: PLoS One. 2025 Sep 4;20(9):e0330157. doi: 10.1371/journal.pone.0330157 (PMC12410808; doi:10.1371/journal.pone.0330157)
Supplement: S1 Table — (PDF) [file pone.0330157.s005.pdf]

de Jong LD, van der Zwaard BC, van Blommenstein MYH, van Loon CJM

Effectiveness and user experiences of a valgus brace in patients with knee osteoarthritis: a mixed-method randomised controlled trial.

Supporting Information Table 1A-E: qualitative data

**Supporting Table 1A** (p2-3):	**Subthemes and quotes emerging from the interview questions about the participants' prior hopes and expectations about (using) the brace**
**Supporting Table 1B** (p4):	**The participants' responses to a question about what they thought when they first saw the brace.**
**Supporting Table 1C** (p5-10):	**Participants' opposing perspectives relating to the perceived influence of using the brace on their body functions, activities and participation.**
**Supporting Table 1D** (p11-20):	**Participants' opposing perspectives relating to the aspects of the valgus brace deemed most relevant in influencing the degree of user satisfaction.**
**Supporting Table 1E** (p21-22):	**Participants' concluding perspectives about their overall experiences and satisfaction with the brace.**

Notes:

In all tables, the "Original verbatim quotes in Dutch" and their *English clean translations* are labelled to indicate the participants' gender (F, M), age (yr), degree of knee osteoarthritis on the Kellgren & Lawrence classification (KL grade 2 or 3) and the number of weeks the brace was used.

The subcodes accompanying the subthemes in Table 1C are derived from the World Health Organization's International Classification of Functioning, Disability and Health.

Supporting Table 1A. Subthemes and quotes emerging from the interview questions about the participants' prior hopes and expectations about (using) the brace.

Theme	Subtheme	Representative quotes
Prior hopes and expectations about (using) the brace	Decrease in knee pain	"Ik had gedacht dat als je 4, 5 dagen de brace om had dat de pijn weg was. Dat je dan even zonder brace weer een paar dagen pijnloos kon lopen."<br>*I had thought that once I'd wear the brace for 4, 5 days that the pain would be gone. And that I could then walk pain-free without the brace for a couple of days.*<br>(M, 60, KL 3, 24 weeks of brace use)
		"[Ik had de verwachting] dat het misschien de pijn een beetje verzacht (…)."<br>*I had the expectation that it would maybe ease the pain a little.*<br>(F, 67, KL 3, 24 weeks of brace use)
		"[Ik had] de verwachting dat het de pijn wat zou verminderen, omdat er wat meer ruimte [in de knie] zou komen."<br>*I had the expectation that it would decrease the pain because it would allow for some more space in my knee.*<br>(F, 75, KL 3, 4 weeks of intermittent brace use)
		"Ik hoopte wel dat (…) de pijn minder werd."<br>*I was hoping that the pain would decrease.*<br>(M, 56, KL 2, 24 weeks of brace use)
		"Ik had wel een beetje hoop (…) dat ik in ieder geval van de pijn af kwam."<br>*I was kind of hoping that I would at least get rid of the pain.*<br>(F, 55, KL 2, 24 weeks of brace use)
		"Ik had verwacht dat de pijn af zou nemen. De slijtage zit aan de binnenkant van mijn knie dus ik dacht dat dat drukken op de buitenkant van mijn knie de pijn zou verminderen."<br>*I expected the pain to decrease. The wear is on the inside of my knee, so I thought that the brace pushing on the outside of my knee would decrease my pain.*<br>(F, 59, KL 3, 8 weeks of brace use)
		"[Ik verwachtte] eigenlijk minder pijn."<br>*I was expecting to have less pain.*<br>(M, 52, KL 3, unknown number of weeks of brace use)
		"[Ik had hoop op] een beetje pijnverlichting."<br>*I was hoping for some pain relief.*<br>(M, 60, KL 2, 24 weeks of brace use)
	Being able to perform the activities as before the knee complaints	"Ik ging hier heel hoopvol naar toe dat ik uitgekozen werd voor een brace, want ik denk 'dat is het!' Ik denk 'dan kan ik lopen en de bezigheden in en om het huis'. (…) [Ik had de verwachting] dat je toch gewoon goed in beweging kan blijven."<br>*I was very hopeful that I would be selected for a brace because I thought 'this is it!' I thought 'then I can walk and do things in and about the house.' I had the expectation that I can still just keep moving well.*<br>(F, 67, KL 3, 24 weeks of brace use)
		"[Ik had ook bepaalde verwachtingen ten aanzien van] traplopen, sporten. Ik ging iedere dag een uur lopen. Dat ging op het laatst pijn doen. Daar had ik hoge verwachtingen van. Dat ik dat gewoon weer op kon pakken."<br>*I also had certain expectations regarding stair climbing, doing sports. I used to walk for an hour every day. That started to hurt in the end. I had high expectations for that. That I could just pick that up again.*<br>(F, 67, KL 3, 24 weeks of brace use)

		"[Ik had verwacht dat] ik weer wat meer zou kunnen wandelen eigenlijk."<br>*I expected to be able to do more walks, actually.*<br>(M, 52, KL 3, unknown number of weeks of brace use)
	Providing knee stability/support	"Ik dacht van 'nou, dat is toch iets stevigs op je been dus dat zou steun moeten kunnen geven'."<br>*I thought 'well, that is something solid on the leg so that ought to be able to provide support.'*<br>(F, 75, KL 3, 4 weeks of intermittent brace use)
		"[Ik had de verwachting] dat hij heel veel meer stevigheid zou geven aan de knie zelf."<br>*I had the expectation that it would provide way more stability to the knee itself.*<br>(M, 51, KL 2, 1 week of brace use)
	Correction of leg axis misalignment	"(…) ik hoop dat ik dan misschien een lichte correctie van dat onderbeen zou hebben."<br>*I hope that maybe I'll have a slight correction of that lower leg.*<br>(F, 74, KL 2, 22 weeks of intermittent brace use)
		"[Ik verwachtte ervan] wat ze zeiden. Die brace is hoofdzakelijk dat die knie niet krom gaat trekken, het been niet krom gaat trekken. Dat je O-benen krijgt."<br>*I expected what they told about it. The brace is mainly to prevent the knee from warping, the leg from warping. So that you don't get bowlegs.*<br>(M, 75, KL 2, 23 weeks of brace use)
	Very specific or general improvements	"Ik had wel een beetje de hoop (…) dat mijn knie niet meer zo vaak op slot zou vallen (…)"<br>*I did have a bit of hope that my knee wouldn't lock up as often.*<br>(F, 55, KL 2, 24 weeks of brace use)
		"Nou, de verwachtingen waren eigenlijk dat het beter zou gaan, dat ik eigenlijk steun eraan zou ondervinden. En dat ik op die manier niet geopereerd zou hoeven worden. (…) Ik had verwacht dat ik eigenlijk wat meer zou kunnen doen. Ik had misschien wel wat hoge verwachtingen (…) ja, dat wanneer ik hem droeg mijn leven wat makkelijker zou worden."<br>*Well, the expectations were that I would be better, that I would find support from it. And that I wouldn't need to undergo surgery because of it. I had expected that I would actually be able to do more. My expectations may have been quite high, yeah, that wearing it would make my life a bit easier.*<br>(M, 52, KL 3, unknown number of weeks of brace use)
		"[Ik hoopte] dat de klachten zou verdwijnen (…)."<br>*I was hoping that the symptoms would dissapear.*<br>(M, 60, KL 2, 24 weeks of brace use)
	No clear expectations	"[Mijn verwachtingen waren] eigenlijk nihil. Geen verwachtingen."<br>*I had no expectations, really. Zero expectations.*<br>(F, 74, KL 2, 22 weeks of intermittent brace use)
		"[Ik had] eigenlijk niet veel [verwachtingen van de brace]. Baat het niet dan schaadt het niet."<br>*I did not expect not much of the brace, really. But if it doesn't help, it doesn't harm.*<br>(M, 60, KL 2, 24 weeks of brace use)

Supporting Table 1B. The participants' responses to a question about what they thought when they first saw the brace.

Theme	Subtheme	Representative quotes
**Aesthetics**	First impression	"Oh, ik word helemaal ingepakt. Ik word een Robocop!' (…) Ik denk 'als hij nog piepen gaat, dan ben ik een robot.' (…) Ja, het is best wel een gevaarte."<br>*Oh my, I'm getting completely wrapped up. I'm becoming a Robocop. I think 'if it starts beeping, then I'm a robot.' Yeah, it's quite a contraption.*<br>(M, 60, KL 3, 24 weeks of brace use)
		"Ik had het gevoel 'dat is voor mij de oplossing.' Dus ik had hem heel graag. Toen ik het ding zag dacht ik 'ja!'"<br>*I felt like 'that's the solution for me.' So I really wanted it. When I saw the thing I thought: 'yes!'*<br>(F, 67, KL 3, 24 weeks of brace use)
		"Toen ik hem zag, o, ja toen die instrumentmaker ermee aankwam, dacht ik wel 'jeetje mina, wat een ding.' (…) Maar dat zakt snel weg."<br>*When I saw it, oh yes, when the orthotist first brought it in, I remember thinking 'wow, the size of that thing!' But that feeling quickly faded.*<br>(F, 74, KL 2, 22 weeks of intermittent brace use)
		"[Mijn eerste gedachte toen ik de brace voor het eerst zag was] Tjonge, wat een ding is dat."<br>*My first thought when I saw the brace for the first time was 'Wow, what a thing that is.'*<br>(F, 75, KL 3, 4 weeks of intermittent brace use)
		"[Toen ik de brace voor het eerst zag, toen dacht ik:] 'best een apparaat.' Ik had het iets compacter verwacht."<br>*When I saw the brace for the first time, I thought 'quite a contraption.' I expected it to be a bit more compact.*<br>(M, 56, KL 2, 24 weeks of brace use)
		"Ik dacht eigenlijk 'dat valt wel mee, dat ding' (…)"<br>*I actually thought 'it's not too bad, that thing.'*<br>(F, 55, KL 2, 24 weeks of brace use)
		"Toen ik hem voor het eerst zag, dacht ik 'o, dat is een grote brace!' Hij zat echt wel van boven tot onder."<br>*When I first saw it I thought 'oh, that's a big brace!' It really covered my leg from top to bottom.*<br>(M, 51, KL 2, 1 week of brace use)
		"Och jee, wat een groot ding. Wat een spektakel. (…) op het werk zeiden ze ook wel 'o, jee, wat heb jij nou aan je been!?' Het is natuurlijk best een groot spektakel wat je om je knie hebt zitten."<br>*Oh my what a big thing. What a spectacle. At work they also said 'Oh dear, what's that on your leg!?' It's quite a spectacle having that around your knee.*<br>(F, 59, KL 3, 8 weeks of brace use)
		"Dat ik dacht 'wat krijgen we nou dan?' […] Groot!"<br>*That I thought 'what's this now?' Big!*<br>(M, 75, KL 2, 23 weeks of brace use)
		"[Toen ik de brace voor het eerst zag toen dacht ik:] 'Wat een groot ding'. Ik had verwacht dat hij een stuk kleiner zou zijn."<br>*When I first saw the brace, I thought 'what a big thing'. I expected it to be a lot smaller.*<br>(M, 52, KL 3, unknown number of weeks of brace use)
		"([…) het is een hele stellage (…) [maar ik vond dat niet vervelend] want ik dacht eerst dat het zo'n elastieken [brace] zou worden. Dan had ik gezegd: '[nee] bedankt.' Want die gaan echt knellen en zeer doen."<br>*It's quite a contraption, but that didn't bother me because at first I thought it would be one of those elastic braces. Then I would have said 'no thanks.' Because those will be really tight and will hurt.*<br>(M, 60, KL 2, 24 weeks of brace use)

Supporting Table 1C. Participants' opposing perspectives relating to the perceived influence of using the valgus brace on their body functions, activities and participation.

Theme	Subtheme	Perceived positive influeces	Perceived neutral and negative influences
**Perceived influence of the brace on body functions**	Pain (b280)	"Hij verlicht wel. Hij haalt 50 procent van de pijn weg."<br>*The brace does alleviate the pain. It takes away 50 percent of the pain.*<br>(M, 60, KL 3, 24 weeks of brace use)	"Helemaal pijnloos is het nooit geweest."<br>*It has never been completely painless.*<br>(M, 60, KL 3, 24 weeks of brace use)
		"Het had [in het begin] best wel succes [op de pijn], maar (…)."<br>*At first it was quite successful in terms of relieving the pain, but...*<br>(F, 67, KL 3, 24 weeks of brace use)	"[de invloed op de pijn] was eigenlijk een beetje van korte duur."<br>*..the influence on the pain was actually a bit short-lived.*<br>(F, 67 , KL 3, 24 weeks of brace use)
		"In het begin merkte ik er niet zo veel van [maar] op een gegeven moment dat hij eigenlijk te veel druk gaf, zeg maar, toen pas merkte ik dat het beter ging."<br>*At first I didn't notice much of it, but when the brace was actually applying too much pressure, that's when I noticed that it was getting better.*<br>(F, 55, KL 2, 24 weeks of brace use)	"Helaas negatief. Dat had ik echt niet verwacht (…) dat ik meer pijn in mijn knie kreeg. Dat is op zich vervelend, maar dat is nog wel te overzien. Maar ik kreeg meer moeite met lopen."<br>*Negative, unfortunately. I really didn't expect to get more pain in my knee. That's annoying in itself, but that was still manageable. But I got more problems with walking.*<br>(F, 75, KL 3, 4 weeks of intermittent brace use)
		"Ik merkte dat hij aan de buitenkant [van mijn knie] iets doet. Dan krijg je aan de binnenkant iets meer ruimte. (…) Dat gaf op een gegeven moment wel dat ik minder steken aan de binnenkant had (…)."<br>*I noticed that it had an effect on the outside of my knee. Then you get a little more space on the inside. At some point that resulted in fewer stabbing pains on the inside.*<br>(M, 51, KL 2, 1 week of brace use)	"Ik werk in de zorg, dus ik loop heel veel, 8 uur, 9 uur per dag zo'n beetje. In het begin dacht ik wel: 'o, het helpt.' Maar nee, ik heb toch het idee dat ik geen verschil merkte. Ondanks dat ik de brace omhad, had ik toch gewoon pijn."<br>*I work in healthcare, so I walk a lot, about 8 hours, 9 hours a day. At first I thought 'oh, it helps.' But no, I still feel like I didn't notice any difference. Despite wearing the brace, I still had pain.*<br>(F, 59, KL 3, 8 weeks of brace use)
		"Hij neemt de pijn niet weg [maar vergeleken met de tijd dat ik de brace niet had is de pijn] minder. Stukken minder. (…) [De pijn tijdens traplopen] is een stuk beter geworden."<br>*It doesn't take the pain away but compared to the time when I didn't have the brace, the pain is less. Much less. The pain during stair climbing has improved significantly.*<br>(M, 75, KL 2, 23 weeks of brace use)	"Als ik de brace droeg dan ging het wel, maar zodra ik de brace weer af deed dan werd het eigenlijk erger."<br>*When I wore the brace, it was okay, but as soon as I took the brace off it actually got worse.*<br>(M, 52, KL 3, unknown number of weeks of brace use)
		"Minder pijn. Veel minder pijn."<br>*Less pain. Much less pain.*<br>(M, 60, KL 2, 24 weeks of brace use)	
	Stability (b715)	"De stabiliteit is wel fijn als je de brace omhebt."<br>*The stability is nice when you're wearing the brace.*<br>(M, 60, KL 3, 24 weeks of brace use)	
		"Het ondersteunt, het geeft stevigheid, (…)."<br>*It supports, it provides stability, ...*<br>(F, 67, KL 3, 24 weeks of brace use)	

		"Misschien [heeft de brace effect gehad op de stabiliteit van mijn knie]. [Ik kon vooral] het verschil merken als die af is."<br>*Maybe the brace has had an effect on the stability of my knee. I especially noticed the difference after taking the brace off.*<br>(F, 74, KL 2, 22 weeks of intermittent brace use)	
		"Ik had wel wat meer stabiliteit (…) Wat steun. Steun aan mijn been, aan mijn knie."<br>*I did have a bit more stability. Some support. Support for my leg, for my knee.*<br>(F, 59, KL 3, 8 weeks of brace use)	
		"In het begin ging ik nog wel eens veel door de knieën heen. Dat is nu stukken minder. Of dat met de brace te maken heeft, weet ik niet, dat zou ik niet durven zeggen. Maar het is in ieder geval een stuk minder geworden. (…) Ik heb er wel steun aan."<br>*Before I used the brace I regularly buckled through my knees. That happens much less now. Whether that's to do with the brace, I don't know, I couldn't say. But that's definitely gotten better. I do find support in it.*<br>(M, 75, KL 2, 23 weeks of brace use)	
		"Ik denk dat als ik hem om had, het wel wat stabieler was."<br>*I think when I had it on it was somewhat more stable.*<br>(M, 52, KL 3, unknown number of weeks of brace use)	
		"Vroeger als ik de trap op ging, dan moest ik niet met links beginnen, want dan lag ik op de trap. Nu kan ik gewoon links beginnen, als hij er omheen zit. Dan heb ik stevigheid."<br>*Earlier, when I went up the stairs I couldn't start with my left leg because then I'd end up lying on the stairs. Now I can start with my left leg when I have the brace on. Then I have stability.*<br>(M, 60, KL 2, 24 weeks of brace use)	
	Mobility (b710)	"[Hoe ver ik mijn knie kan buigen en strekken is] precies hetzelfde. Dat gaat goed."<br>*The degree to which I can bend and straighten my knee is exactly the same. That's going well.*<br>(M, 75, KL 2, 23 weeks of brace use)	"Je bent wel beperkter met een brace aan (…) met autorijden. Op de fiets niet, maar autorijden, zitten bijvoorbeeld in de bioscoop of zo. Je kunt je been niet zetten zoals je normaal zet. Daar word je wel in gehinderd. Dat is wel jammer."<br>*You are somewhat restricted while wearing the brace when driving a car. Not on the bike, but when driving the car or sitting in the cinema for example. You can't position your leg like you normally do. You are limited in that aspect. That's a pity.*<br>(M, 60, KL 3, 24 weeks of brace use)
		"Nee [het heeft geen invloed op de beweeglijkheid van mijn knie]."<br>*No, it has no influence on the mobility of my knee.*<br>(M, 60, KL 2, 24 weeks of brace use)	

	Muscle power (b730 & b749)	"[Invloed op] de kracht [had de brace] zeker niet, denk ik."<br>*The brace definitely did not have any influence on my leg strength, I think.*<br>(F, 75, KL 3, 4 weeks of intermittent brace use)	
		"Nee [de brace heeft geen invloed gehad op de kracht van mijn knie] (…)"<br>*No, the brace did not have any influence on the strength of my knee.*<br>(F, 55, KL 2, 24 weeks of brace use)	"Ik heb [wel] het idee dat mijn been, de spiermassa minder is geworden […] want mijn been is slanker."<br>*However, I do feel that the muscle mass has decreased because my leg is slimmer.*<br>(F, 55, KL 2, 24 weeks of brace use)
		"[Of de brace effect heeft gehad op de kracht van mijn been] Dat weet ik niet. (…) [maar ik heb ook het idee dat mijn been] niet minder [sterk is geworden door het gebruik van de brace]."<br>*I don't know if the brace has had an effect on the strength of my leg, but I also think that my leg hasn't gotten any weaker from using it.*<br>(M, 75, KL 2, 23 weeks of brace use)	"Ik kwam daar en zegt [de sporttherapeute] 'die spieren, je kunt merken dat ze al dun worden.' Dus die brace vangt wel heel veel op, maar die spieren gaan steeds minder doen."<br>*I went there and the sports therapist said 'those muscles, you can tell they're getting thinner.' So, that brace does absorb a lot, but those muscles are becoming less active.*<br>(M, 51, KL 2, 1 week of brace use)
		"Nee [ik heb geen effect op de kracht in mijn been gemerkt], hij is al sterk. Ik had meer het idee dat het ook door de oefeningen kwam die ik van de fysiotherapeut kreeg."<br>*No, I haven't noticed any effects on the strength of my leg, it's strong as it is. I felt more like that it was also due to the exercises that I got from the physiotherapist.*<br>(M, 52, KL 3, unknown number of weeks of brace use)	
	Control of voluntary movements (b760)	"Met de brace is het [korte bochten maken met het been] dan beter, omdat je dan die soepele draaiing niet echt kan maken, omdat die stevig is. (…) Dan heb je ook minder pijn."<br>*With the brace it's easier to make sharp turns with the leg because the brace is sturdy and it doesn't allow for rotation in the knee. Then you also have less pain.*<br>(M, 56, KL 2, 24 weeks of brace use)	"Ja absoluut, [ik moest mijn bewegingsgedrag bij traplopen en de stoep op en afstappen door de brace aanpassen]."<br>*Yes definitely, I had to adjust my movement behavior when climbing the stairs and stepping up and down the curb due to the brace.*<br>(F, 67, KL 3, 24 weeks of brace use)
		"Voor die tijd was het draaien van de knie, mijn been draaien, dat is nu ook bijna weg, met de brace om. Dus die steun heb ik dus wel."<br>*The awkward twisting of the knee when turning around is almost gone now with the brace on. So, I do have that support.*<br>(M, 75, KL 2, 23 weeks of brace use)	
		"De oefeningen [bij de fysiotherapeut] waren wel makkelijker met brace. (…) dan had ik meer kracht en stabiliteit als ik die brace omhad. Ja, dus als ik dan zo nadenk, (…) dat ging wel wat beter. Wat minder pijnlijk."<br>*The physiotherapy exercises were easier with the brace on. That's when I had more strength and stability. So yeah, when I think about it, that went a bit better. A bit less painful.*<br>(M, 52, KL 3, unknown number of weeks of brace use)	

	Confidence (b1266)	"Als ik de brace om had, ging die eigenlijk niet meer op slot. Dus die winst had het sowieso als de brace om was. Dus het vertrouwen in mijn knie in mijn dagelijkse werk kwam weer heel langzamerhand terug. Als ik hem niet om heb, vertrouw ik gewoon niet op mijn knieën."<br>*When I was wearing the brace my knee actually no longer locked. So that was a gain anyway when the brace was on. So the confidence in my knee during my daily work gradually returned. When I don't have it on, I just don't trust my knees.*<br>(F, 55, KL 2, 24 weeks of brace use)	
	Functions of lymphatic vessels (b4352)	"Als hij af is, dan merk je binnen twee uur een dikke knie. Dan doet het zeer. Als hij om is, dan is het prima."<br>*When the brace was off I noticed a swollen knee within two hours. That's when it hurts. When the brace is on, it's fine.*<br>(M, 60, KL 2, 24 weeks of brace use)	
**Perceived influence of the brace on activities**	Walking and moving (d450|)<br><br>Walking long distances (d4500),<br><br>Walking on different surfaces (d4502)<br><br>Going up and down stairs (d451)<br><br>Climbing (d4551)	"Gewoon lopen, daar ondersteunt hij in. Dat is wel prettig. (…) Als ik veel lopen moest, dan deed ik hem aan."<br>*Normal walking, the brace supports that. That's quite nice. When I had to walk a lot I would put it on.*<br>(M, 60, KL 3, 24 weeks of brace use)	"[…] eigenlijk heb ik er [bij het lopen] geen baat bij gehad"<br>*In terms of pain I haven't really benefitted from the brace during walking.*<br>(F, 67, KL 3, 24 weeks of brace use)
		"Ik hoefde hem [van de instrumentmaker] alleen [tijdens activiteiten als lopen, traplopen, tuinieren, sporten] te dragen, maar ik ben hem bijna hele dagen gaan dragen. (…) Zonder brace geef je [die activiteiten] eerder op."<br>*The orthotist advised me to only wear the brace during activities like walking, stair climbing, gardening, exercising, but I started wearing it almost all day. Without the brace, you tend to give up on those activities sooner.*<br>(F, 67, KL 3, 24 weeks of brace use)	"[Traplopen en lopen op ongelijke ondergrond] werd niet makkelijker door die brace. (…) Nee, [verbeteringen in die activiteiten] heb ik niet gemerkt."<br>*Stair climbing and walking on uneven ground did not become easier with that brace. No, I did not notice any improvements in those activities.*<br>(F, 75, KL 3, 4 weeks of intermittent brace use)
		"Ja, bij het wandelen (…) ik ga op zaterdag naar de markt in Arnhem. En dan met de bus en dan doe ik hem ook om tijdens winkelen, (…) Als ik zelf niet hoeft te rijden, dan doe ik hem om. (…) ik draag hem bij het wandelen en het winkelen."<br>*Yes, when walking. On Saturdays I go to the market in Arnhem. And then I take the bus and I also put the brace on while shopping. When I don't have to drive myself, then I put it on. So, I wear it during walking and shopping.*<br>(F, 74, KL 2, 22 weeks of intermittent brace use)	
		"[Uiteindelijk heb ik de brace het meeste gedragen tijdens] Lopen. (…) Ik loop [bij het dragen van de brace] wel soepeler de trap af. Dus dat vertrouwen komt wel meer terug."<br>*Ultimately I wore the brace the most during walking. I do walk down the stairs more easily when wearing the brace. So, that confidence does come back more.*<br>(F, 55, KL 2, 24 weeks of brace use)	

		"[De belangrijkste reden voor mij om de brace te gaan dragen waren voor] het lopen, traplopen, gewoon lopen."<br>*The most important reason for me to start wearing the brace was for walking, stair climbing, just walking.*<br>(F, 59, KL 3, 8 weeks of brace use)	
		"[Ik heb de brace het meeste gedragen tijdens] Tuinieren en lopen. (…) Ik kan nu langer lopen. Als ik hem niet om heb, dan heb ik constante pijn."<br>*I wore the brace most during gardening and walking. I can walk longer now. When I don't wear it I have continuous pain.*<br>(M, 75, KL 2, 23 weeks of brace use)	
		"Ik moet over een hekje stappen als ik mijn tuin in wil. Met brace kun je toch iets makkelijker over het hekje stappen. Het is nog geen gezicht, moet ik zeggen, maar het lukt wel."<br>*I have a small fence at the entrance of my garden. With the brace on I can can step over this fence just a bit more easily. It looks ridiculous I must say, but at least it works.*<br>(F, 67, KL 3, 24 weeks of brace use)	
	Lifting and carrying objects (d430)	"[Bij kleine stukjes tillen] Dan helpt hij wel een beetje, ja, omdat hij dan de been een beetje naar binnen duwt, heb ik ook iets minder druk erop."<br>*It does help a bit during lifting things short distances. Because the brace slightly pushes the leg inward I also have a little less pressure on it.*<br>(M, 56, KL 2, 24 weeks of brace use)	
		"Omdat ik wat beter kon wandelen met die brace. Ik wandelde nogal graag, doe ik nu al een tijdje niet. (…) En het werken in de tuin, daar heb ik hem wel steeds bij gebruikt, dat ging op zich wel goed. Dan had ik wel meer stabiliteit als ik bijvoorbeeld met de kruiwagen liep (…) Als ik dus wat [zwaardere activiteiten] deed dan ging het met brace wat makkelijker dan zonder brace."<br>*Because I could walk a bit better with that brace. I used to enjoy walking a lot, but I haven't been doing that for a while now. And I always used it when working in the garden, that went quite well. Then I had more stability, for example when I was walking with the wheelbarrow. So, when I was doing some heavier activities it was easier with the brace than without it.*<br>(M, 52, KL 3, unknown number of weeks of brace use)	
	Kneeling (d4102)		"Ik werk in de zorg dus ik zit vaak op mijn knieën. Dat ging gewoon niet. Ik kon niet echt knielen met de brace om."<br>*I work in healthcare so I'm on my knees often. That just didn't work. I couldn't really kneel with the brace on.*<br>(F, 59, KL 3, 8 weeks of brace use)

			"(…) zitten op de knieën, dat ging wat moeilijker, omdat [de brace] dan ook in de weg zat."<br>*Kneeling down on my knees was a bit more difficult because then the brace got in the way.*<br>(M, 52, KL 3, unknown number of weeks of brace use)
	Moving around using transportation (d4708)	"[de goede stabiliteit merkte ik ook tijdens het] Fietsen. Dat was met rechts een beetje en dan links er achteraan. Nou kan je gewoon doortrappen."<br><br>*I also noticed good stability during cycling. It used to be pushing with one leg and the other was just hanging on. Now I can just keep pedaling with both.*<br><br>(M, 60, KL 2, 24 weeks of brace use)	"Met autorijden. Op de fiets niet, maar autorijden, (…) Ik zit op de heftruck en dat soort dingen. Met op- en afstappen van de heftruck. (…) Je kunt de been niet zetten zoals je normaal zet. Daar word je wel in gehinderd, dat is wel jammer."<br>*"When driving a car. Not on the bicycle, but driving the car. I operate a forklift and things like that. When getting on and off the forklift you can't position the leg like you normally do. That's a hinderance, that's a pity.*<br>(M, 60, KL 3, 24 weeks of brace use)
			"Alleen fietsen ging wel vervelender, omdat hij dan in de weg zit. (…)"<br>*Only cycling went worse because then the brace is in the way.*<br>(M, 52, KL 3, unknown number of weeks of brace use)
**Perceived influence of the brace on participation**	Remunerative employment (d850)	"Dat ik gewoon mijn werk kan blijven doen, daar heeft de brace wel mee geholpen. (…) Dus nu doe ik bewegingspelletjes met de kinderen mee. Daar moet ik dan aanpassingen in maken, maar dat gaat nu beter."<br>*The brace did enable me to keep doing my job. So now I participate in playing games with the children, I do have to make some adjustments for that, but that's going better now.*<br>(F, 55, KL 2, 24 weeks of brace use)	"Voor mij heeft het geen zin. Vooral niet in mijn werk. Het belemmerde op een gegeven in mijn werk [tijdens] Staan, bukken, zitten. Eigenlijk alles."<br><br>*It's not useful for me. Especially not during my work. At some point, using the brace started to hinder me in my work during standing, bending over, sitting. Everything, basically.*<br>(M, 51, KL 2, 1 week of brace use)

Supporting Table 1D. Participants' opposing perspectives relating to the aspects of the valgus brace deemed most relevant in influencing the degree of user satisfaction.

Theme	Subtheme	Perceived positive user experiences	Perceived neutral and negative user experiences
**User satisfaction**	Simplicity of use (in terms of using, learning to use and donning and doffing the brace)	"In het begin moet je [bij het aandoen] even aftasten hoe hij moet zitten, hoe strak. Op een gegeven moment voel je dat. (…) ik heb er geen moeite mee."<br>*At first I had to figure out how tight the brace should be when putting it on. At some point you'll get a feel for that. I don't have any trouble with it.*<br>(M, 60, KL 3, 24 weeks of brace use)	"Ik kan [de brace] erop leggen, maar ik kom (…) niet bij [de onderste banden van de brace]. (…) [Ik had de meeste moeite met] De clipjes aan de zijkant. (…) Voor mij alleen zal het wel moeilijk zijn om hem goed om krijgen. Dat vind ik wel een nadeel. (…) Ik vind het voor iemand alleen een beetje gebruiksonvriendelijk."<br>*I can position the brace on my leg, but I can't reach the lower straps. I was struggling most with the clips on the side. It will be difficult to put it on correctly on my own. I find that a disadvantage. I think it's a bit user-unfriendly for someone alone.*<br>(F, 74, KL 2, 22 weeks of intermittent brace use)
		"In het begin was het een probleem, want toen werd gezegd: 'je moet hem onder je kleren aan.' (…) Onder de kleren was enorm lastig. Later bleek dat je hem over de kleren kon dragen. Dat had ik heel snel op slag. Het gebruik ervan was geen probleem, het aan- en uitdoen."<br>*At first, putting the brace on and taking it off it was a problem, because they said 'you have to wear it under your clothes.' Wearing it under my clothes was extremely difficult. But later it turned out that you could wear it on top of your clothes. I soon became very efficient at that. Using the brace was not a problem, putting it on and taking it off.*<br>(F, 67, KL 3, 24 weeks of brace use)	"[Het aan en uitdoen] Dat kreeg ze zelf niet voor elkaar."<br>*She couldn't manage to put it on and take it off by herself.*<br>(Spouse of F, 74, KL 2, 22 weeks of intermittent brace use)
		"[Het aan- en uitdoen] ging prima. […] Ja, het ging eigenlijk heel makkelijk. Ja, er is voorgedaan hoe die aan moet en hoe die stevig het beste zit. En, dat ging eigenlijk vanzelf."<br>*Putting it on and taking it off went fine. Yes, it actually was quite easy. Yes, they showed me how to put it on and that it fits best when it is tight. And that went smoothly, actually.*<br>(F, 55, KL 2, 24 weeks of brace use)	"Ik vind [het aan- en uitrekken van de brace] behoorlijk lastig voordat je het gevoel hebt dat hij goed zit. In het begin stond het nog keurig op mijn been afgetekend waar hij moet zitten. (…) Maar dan nog is het lastig om hem echt net op de goede plek te krijgen. Dat vind ik echt lastig. (…) Je voelt het wel op een gegeven ogenblik, maar het heeft toch wel tijd nodig voordat je hem goed hebt. Ik vind het in- en uitstappen uit al die [banden] (…) ook lastig. (…) De handzaamheid is gering. (…) Ik vind hem dus gewoon lomp en heel onhandig."<br>*I find putting the brace on and taking it off quite difficult before you get this feeling of it fitting properly. At first the proper position was neatly marked on my leg. But even then it's still difficult to get it in exactly the right position. I find that really difficult. You do feel that at some point, but it takes time before you get it right. I found getting in and out of all those straps also difficult. The convenience of handling is limited. I just find it very inconvenient to use.*<br>(F, 75, KL 3, 4 weeks of intermittent brace use)
		"(…) het was heel gemakkelijk om [de brace] aan en uit te trekken. (…) Ermee leren omgaan ging heel makkelijk. Ik heb uitleg gehad hoe ik hem moest doen. Dat was heel simpel. (…) ik vond het gebruiksgemak prima."	"[Het leren met de brace omgaan] was even lastig, met hoe strak hij zit, de druk en alles. (…) Dan moest hij echt na een uurtje of 5 eraf. Dan begon het gewoon zeer te doen."

		It was very easy to put the brace on and take it off. Learning to use it was very easy. I got instructions on how to put it on. That was very easy. I found the ease of use excellent.<br>(F, 59, KL 3, 8 weeks of brace use)	*Learning to use the brace was a bit difficult at first, with how tight it is, the pressure and all. I really had take it off after about five hours. Then it just started to hurt.*<br>(M, 56, KL 2, 24 weeks of brace use)
		"In het begin was het even puzzelen. (…) [Na ongeveer twee weken dacht ik:] 'Nou heb ik hem goed in de vingers.'"<br>*At first it was a bit of a puzzle. After about two weeks I thought 'now I've got the hang of it.'*<br>(M, 75, KL 2, 23 weeks of brace use)	
		"[het leren] aan en uit doen van de brace [werd makkelijker] na een week."<br>*Learning to put the brace on and take it off got easier after a week.*<br>(M, 52, KL 3, unknown number of weeks of brace use)	
		"[Het aan- en uitdoen] is vrij simpel. (…) [Het leren omgaan met de brace ging] Bijna automatisch. (…) De eerste twee, drie dagen was het even bijstellen, trekken. Dat het te strak zat of zo, maar ik heb er geen hinder van."<br>*Putting it on and taking it off is quite easy. Learning to use the brace almost went automatic. The first two, three days required some adjusting and pulling. That it was too tight or something, but I didn't bother me.*<br>(M, 60, KL 2, 24 weeks of brace use)	
	Dimensions<br><br>(i.e. the brace's fit in terms of convenience of its height, width and length)	"De pasvorm is heel goed. Hij zit als gegoten. Als je hem zelf een beetje afstelt, dan is het perfect."<br><br>*The brace's fit was very good. It fitted like a glove. When you adjusting it yourself it is perfect.*<br><br>(M, 60, KL 3, 24 weeks of brace use)	"[Ik merkte] Dat ik heel vaak met mijn knie ergens tegenaan botste. Thuis tegen de tafel, (…) Eén keer tegen de deur, dat ik dacht van: 'o, gut ja, daar zit een deur.' (…) Het is maar twee centimeter of zo, zo'n knop, maar toch is het anders."<br>*I noticed that I regularly bumped my knee against things. At home, against the table. Once against the door, and then I thought 'Oh, right, there's a door there.' That hinge on the side is just two centimeters or so, but it's different.*<br>(F, 75, KL 3, 4 weeks of intermittent brace use)
		"Hij zat wel goed. Dit was lekker zacht, dus daar had je verder helemaal geen last van, die kussentjes. […] Ja, op zich was [het pascomfort] wel goed."<br>*It fitted allright. This was nice and soft, so I didn't have any trouble with those pads. Yeah, overall, the fit was quite good.*<br>(F, 75, KL 3, 4 weeks of intermittent brace use) KL 3	"Dus die achterkant kwam bijna in mijn knieholte, waardoor ik bijna niet meer kon bewegen. Dan moest ik hem even omhoog trekken."<br>*So, the back strap was almost sitting in knee pit which made it almost impossible for me to move. Then I had to pull the brace up a bit.*<br>(M, 52, KL 3, unknown number of weeks of brace use)
		"Ja, die [pasvorm] zit mij wel goed. Wat ik zeg, af en toe moet ik denken van 'o ja, moet hij moet los en wat opgeschoven worden.' Maar voor de rest zit die goed."<br>*Yeah, the brace fits me well. Like I said, sometimes I have to remember to adjust it a bit and slide it up. But other than that, it fits well.*<br>(F, 55, KL 2, 24 weeks of brace use)	
		"Op het moment dat hij goed zit, dan heb je er weinig last van, want hij zit goed aan alle kanten."<br>*Once the brace fits well it doesn'n bother you much because it fits well on all sides.*	

		(M, 51, KL 2, 1 week of brace use)	
		“[De pasvorm was] Goed, prima.”<br>*The brace’s fit was good, fine.*<br>(M, 75, KL 2, 23 weeks of brace use)	
		“[Over het comfort van de brace ben ik] Tevreden. [Op een schaal van 1 tot 10 geef ik een] Zeven.”<br>*I’m satisfied about the brace’s comfort. On a scale of one to ten I’d grade it a seven.*<br>(M, 52, KL 3, unknown number of weeks of brace use)	
		“Hij zakte af en toe wel eens af, maar dat is gewoon even aantrekken. Net zoals nou met dat warme weer. Dan begin je te zweten, dan gaan die banden een beetje [zakken].”<br>*The brace did slip down occasionally, but that's just a matter of pulling it up again. Just like now, with this warm weather. You start sweating and then the straps tend to slip a bit.*<br>(M, 60, KL 2, 24 weeks of brace use)	
	Comfort (in terms of physical complications)	“Nee [ik heb geen fysieke klachten ervaren als gevolg van het dragen van de brace]. (…)”<br>*No, I did not experience any physical complaints as a result of wearing the brace.*<br>(M, 60, KL 3, 24 weeks of brace use)	“Op de blote huid is het bijna niet te doen. Vooral niet als het warm is. Dan gaat hij zweten en jeuken en (…) hij gaat schuren. Dan krijg je schaafwonden.”<br>*It's almost undoable having the brace on bare skin. Especially when it's hot. Then it starts sweating and itching and chafing. That's when you get abrasions*<br>(M, 60, KL 3, 24 weeks of brace use)
		“Nee, [huidirritatie of drukplekjes] helemaal niet gehad. Maar ik droeg hem ook op de broek.”<br>*No, no skin irritation or pressure sores at all. But then again, I wore it over my pants.*<br>(M, 52, KL 3, unknown number of weeks of brace use)	“Het werd rood [onder de kniepads] en het ging pijn doen. Toen kon ik gelijk komen en nieuwe pads werden besteld. Er zijn nieuwe pads op gezet en toen ging het goed.”<br>*It turned red underneath the kneepads and it started to hurt. Then I could come right away and new pads were ordered. They put on some new pads and then it was fine.*<br>(F, 67, KL 3, 24 weeks of brace use)
			“[Ik heb hem ook tijdens het sporten gedragen,] Dus ook op het blote been. En dat gaat van hoep en hoep en die kant en die kant met als gevolg dat ik zeg maar drie soorten snijwonden in de knieholte of bij de knieholte had. En toen zijn die [bandkussentjes] veranderd. Maar ik heb hem nooit meer op [de blote huid] gedragen. (…) Nee, [ik heb daarna nooit meer last gehad van huidirritaties of drukplekjes].”<br>*I also wore it during sporting activities, so on my bare skin. And then it shifts from here to there and everywhere, resulting in like three kinds of lacerations in or near the back of my knee. And then those pads were changed. But I never wore it on my bare skin again. No, I’ve not experienced any skin irritations or pressure sores after that.*<br>(F, 74, KL 2, 22 weeks of intermittent brace use)

			"[Ik kreeg] Een wondje [op de laterale condyl], ja. Niet vreselijk, hoor."<br>*I got a little wound on the lateral condyle, yeah. Not terrible, though.*<br>(F, 75, KL 3, 4 weeks of intermittent brace use)
			"Ik heb hem ook een poosje onder de broek gedragen. Dan kreeg ik een beetje dat het rood uitsloeg, de huid. (…) Hetgeen wat ik toen had op het blote been, dat had ik (…) niet [bij het dragen van de brace over de broek]."<br>*I also wore the brace under my pants for a while. Then I got a bit of a red rasch on my skin. I didn't have that then wearing the brace over my pants.*<br>61 / (M, 56, KL 2, 24 weeks of brace use)
			"Het is geen lekker katoen wat tegen je huid aanzit, zeg maar. Dus [bij het dragen op de blote huid] broeit dan op een gegeven moment."<br>*It's not like there's a nice piece of cotton against your skin. So when wearing the brace on bare skin it starts to fester at some point.*<br>62 / (F, 55, KL 2, 24 weeks of brace use)
			"Nou ja, wat ik wel heb is, aan het einde van de dag is er een deukje achter in mijn been [maar] In de loop van de nacht trekt dat wel bij."<br>*Well, what I do have is, at the end of the day there's a little indentation at the back of my leg. But that will clear overnight.*<br>(F, 55, KL 2, 24 weeks of brace use)
			"(…) in begin had ik dat wondje. Later had ik daar een elastische kous om heen."<br>*I had that little wound at first. Later I wore a compression stocking around it.*<br>(M, 51, KL 2, 1 week of brace use)
			"Geen drukplekken, geen blaren of weet ik veel wat. (…) Nee, alleen de eerste dag (…) drukte hij heel erg op de binnenkant van mijn knie. Toen had ik echt hele erge pijn en toen heb ik hem af gedaan. (…) Dat is de enige klacht die ik ervan heb gehad, de eerste avond."<br>*No pressure sores, no blisters or anything like that. No, just the first day it was pushing on the inside of my knee quite hard. Then I had some really severe pain and then I took it off. That's the only problem I've had with it, that first evening.*<br>(F, 59, KL 3, 8 weeks of brace use)
			"Hierachter [de knie] had ik [een] wondje (…) [maar] dat was ook zo weer weg."<br>*I had a little wound behind my knee, but that disappeared quite quickly.*<br>(M, 75, KL 2, 23 weeks of brace use)
			"Een klein wondje aan de binnenkant van de knie."<br>*A small wound on the inside of my knee.*<br>(M, 60, KL 2, 24 weeks of brace use)

	Comfort (in terms of psychological well-being)	" [Het dragen van de brace is] helemaal niet [van invloed geweest op mijn zelfbeeld en heeft niet geleid tot gevoelens van schaamte]."<br>*Wearing the brace has not affected my self-image at all and has not led to feelings of shame.*<br>(M, 60, KL 3, 24 weeks of brace use)	"Ik had een beetje schaamte. 'Dat is toch geen gezicht zo over die broek, draag hem er maar onder.' Maar ik was er gelijk van genezen toen [de klachten bij het op de blote huid dragen] eruit kwam. (…) Ik heb totaal geen gêne meer als ze met dat ding loopt. Ik vind het af en toe nog wel stoer…"<br>*I felt a bit ashamed. It just looks ridiculous wearing them on top of the pants like that. Just wear it underneath!. But I was soon cured of that feeling when her physical complications came to light. Now I don't feel any embarrassment at all anymore when she's walking around with that thing. In fact, sometimes I think it is kind of cool…*<br>(Spouse of F, 74, KL 2, 22 weeks of intermittent brace use)
		"Nooit, geen minuut [geschaamd voor de brace]. (…) Nee, [het dragen van de brace heeft geen invloed gehad op het beeld dat ik van mezelf heb,] je went er heel snel aan. Mensen kijken, of er worden vragen over gesteld. Dan denk ik: 'ja, het hoort gewoon bij mij.'"<br>*Never, not one minute have I felt ashamed of the brace. No, wearing the brace hasn't affected the image I have of myself, you get used to it very quickly. People look or ask questions about it. And then I think 'yeah, it's just part of me'.*<br>(F, 67, KL 3, 24 weeks of brace use)	"Nee, [ik schaamde me er niet voor de brace want hij] zat onder mijn broek. Dus ik heb alleen maar wijde broeken aangehad. (…) Hij kan er ook overheen, maar dat vind ik zo'n dom gezicht."<br>*No, I wasn't ashamed of the brace because it was under my pants. So I only wore loose pants. I can put it over my pants allright, but I think that just looks silly."*<br>(F, 75, KL 3, 4 weeks of intermittent brace use)
		"Nee, helemaal niet [voor geschaamd]."<br>*No, I have not felt ashamed at all.*<br>(F, 74, KL 2, 22 weeks of intermittent brace use)	"In het begin [vond ik het] wel een klein beetje [vervelend dat andere mensen er naar kijken]. (…) Nee [ik schaamde me er niet voor], dat niet. Anders was ik er ook niet aan begonnen."<br>*At first, I did find it a little annoying that other people were looking at it. But no, I wasn't ashamed of it. If so, I wouldn't have signed up for it.*<br>(M, 56, KL 2, 24 weeks of brace use)
		"Nee [geen schaamte of gene voor gehad]. (…) De kinderen [hebben er wel opmerkingen over gemaakt want] die voelen het als ze tegen je aan liggen. Dat ligt niet lekker."<br>*No, no feelings of shame or embarrassment about it. The children did make comments about it because they can feel the brace when they're snuggled up against you. That doesn't feel comfortable.*<br>(F, 55, KL 2, 24 weeks of brace use)	
		"Als het voor je gezondheid helpt, vind ik het niet erg [dat de omgeving hem ziet]."<br>*If it is good for your health I don't mind that other people see it.*<br>(M, 51, KL 2, 1 week of brace use)	
		"Nee [ik vond het niet vervelend dat andere mensen er wat van zeiden], want ik denk 'als het me helpt, wat maakt het uit.' (…) Nee, hoor [geen gevoelens van schaamte]. Het is niet anders. Wat dat betreft, sta ik daar heel nuchter in. Als het me helpt, wat maakt het dan uit hoe het eruit ziet. Ja, toch? Het gaat om het resultaat."	

		No, I didn't bother me that other people commented on it, because I feel that 'if it helps me, what does it matter.' No, no feelings of shame at all. It is what it is. As far as that's concerned I'm very pragmatic. If it helps me, who cares how it looks, right? It's all about the result.<br>(F, 59, KL 3, 8 weeks of brace use)	
		"Ik had wel het idee dat mensen naar me keken. (…) Nou, ik ben zanger van beroep, dus ik ben wel gewend dat mensen naar me kijken. Het doet me niet zoveel als mensen naar me kijken. (…) Nee [geen invloed gehad op de draagtijd van de brace of het beeld wat ik van mezelf heb]."<br>*I did feel like people were looking at me. Well, I'm a professional singer by trade so I'm used to people looking at me. It doesn't bother me much if people look at me. No, it didn't affect the wearing time of the brace or the image I have of myself.*<br>(M, 52, KL 3, unknown number of weeks of brace use)	
		"Nee [ik schaamde mij er niet voor]."<br>*No, I did not feel ashamed about it.*<br>(M, 60, KL 2, 24 weeks of brace use)	
	Adjustments (i.e. simplicity in setting and fixing the components of the brace)	"Ja, [de brace] heeft genoeg [instel]mogelijkheden met het inbussleuteltje of met de banden. (…) ik heb hem langzaamaan strakker gedaan zodat de knie wel naar binnen drukte."<br>*Yes, the brace has plenty of adjustment options with the Allen key or with the straps. I gradually tightened it so that the knee was pushing inwards.*<br>(M, 60, KL 3, 24 weeks of brace use)	"Ik vond hem ook heel erg moeilijk [in te stellen]. Want iedere keer als ik dan weer [bij de instrumentmaker] terugkwam, dat ik dacht dat hij goed zat en dan (…) bleek dat die toch niet goed zat. Dus hij was wel vrij moeilijk in te stellen. (…) op het laatste leerde ik er beter mee omgaan, ook met het omdoen en zo. In het begin was het gewoon vervelend. Moest je echt mee leren werken. Op het laatst ging het wel redelijk. (…) Ik denk een week of drie [dat het duurde om het leren instellen van de brace onder de knie te krijgen]"<br>*I also found the brace very difficult to set. Because every time I came back to the orthotist I thought it fitted right and then it turned out it didn't. So it was quite difficult to set. Later on I got better at using it and putting it on and such. At first, it was just annoying. You really had to learn to work with it. Towards the end it went reasonably well. I think it took about three weeks to get the hang of setting the brace right.*<br>(M, 52, KL 3, unknown number of weeks of brace use)
		"Met die banden ben ik natuurlijk altijd bezig. Als ik hem dicht moet maken, dan maak ik aan de achterkant die bovenste wel los. Dan zit het beter op zijn plek. Ik heb die schroefjes wel eens strakker moeten draaien dat ik dacht dat het niet meer steunde. Dat het niet meer aansluit op mijn knie. Dan draaide ik hem iets. (…) Ja, op dat moment had je wel het gevoel dat je goed bezig was."<br>*I'm always working with those straps. When I have to close the brace I just loosen the top one at the back. Then it's positioned better. I've had to tighten those screws every now and then when I thought the brace wasn't supportive enough anymore,*	

		when it wasn't sitting snugly around my knee. Then I turned it a bit. Yeah, at that point I felt like I was doing the right thing.<br>(F, 67, KL 3, 24 weeks of brace use)	
		"[Qua instelmogelijkheden was hij] goed. (…) Met die klittenband trek je hem open en dan doe je hem losser. Als hij los zit, dan doe je hem iets strakker. Als je de sleutel bij hebt, dan kun je het makkelijker strakker zetten."<br>*In terms of adjustment options it was good. With those Velcro straps you open it up and then you loosen the brace. If it's too loose, you just strap it in a bit more. If you have the Allen key with you, you can tighten it more easily.*<br>(M, 56, KL 2, 24 weeks of brace use)	
		"(…) ik heb hem af en toe wat strakker gezet, (…) En met een kledingwissel de banden iets strakker."<br>*Every now and then I tightened the brace a bit. And when changing clothes I tightened the straps slightly.*<br>(F, 55, KL 2, 24 weeks of brace use)	
		"Ja, [ik gebruikte de instelmogelijkheden] alleen als ik hem aandeed. Als ik hem aandeed, dan had ik even die klittenbandjes wat wijder om hem makkelijk dicht te klikken. Dan trok ik hem vast, totdat ik zelf het gevoel heb van 'hij zit goed strak nu.'"<br>*Yes, I used the adjustment options only when I was putting the brace on. When I put it on, I would loosen those Velcro straps just a bit to easily close the brace. Then I would tighten it until I felt like 'it's snug now.'*<br>(F, 59, KL 3, 8 weeks of brace use)	
		"[Het spelen met de instellingsmogelijkheden van de brace] gaat geweldig."<br>*Fiddling with the adjustment options of the brace is going great.*<br>(M, 75, KL 2, 23 weeks of brace use)	
		"Ja, [over de instelmogelijkheden ben ik redelijk tevreden (…) Als ik een cijfer mag geven, denk ik een zeven, dus voldoende."<br>*"Yes, I'm fairly satisfied about the adjustment options. If I had to grade it, probably a seven out of ten, so satisfactory.*<br>(M, 52, KL 3, unknown number of weeks of brace use)	
	Durability (i.e. robustness and sturdiness of the brace;<br>in terms of structural	"Hij is lekker stevig als je hem goed aandoet. (…) Nee, het is een stevig ding."<br>*It feels solid when you put it on properly. No, it's a solid thing.*<br>(M, 60, KL 3, 24 weeks of brace use)	"Je blijft (…) wel eens achter [het clipje aan de zijkant] haken dat hij losspringt."<br>*You occasionally get caught behind that clip on the side, causing the brace to come loose.*<br>(F, 67, KL 3, 24 weeks of brace use)
		"[Mijn indruk van de robuustheid en de stevigheid van de brace] was goed. Voor mijn gevoel, en vooral in het begin, deed hij wat hij moest doen. Ik had meer steun met lopen. (…) Nee [de brace is niet kapot geweest]."	"Nee [de brace is nooit beschadigd geraakt of kapotgegaan]. (…) Ik ben wel van die [clipjes waar de banden doorheen lopen] kwijtgeraakt."

	strength, construction and breakages and damages)	*I got a good impression of the robustness and sturdiness of the brace. In my opinion, and especially in the beginning, it did what it was supposed to do. It gave me more support while walking. No, the brace has never been damaged.*<br>(F, 67, KL 3, 24 weeks of brace use)	*No, the brace has never been damaged or broken. I did lose some of those clips where the straps go through.*<br><br>(F, 75, KL 3, 4 weeks of intermittent brace use)
		"Ja, voor mijn gevoel is het een stevig ding."<br><br>*Yeah, to me it feels like a solid thing.*<br><br>(F, 74, KL 2, 22 weeks of intermittent brace use)	"Door mijn werkzaamheden zakte hij af. Die banden sprongen los in die clips. (…) Af en toe schoten die banden los."<br>*Due to my work activities it slipped down. The straps came loose in those clips. Every now and then those straps would come loose.*<br>(M, 51, KL 2, 1 week of brace use)
		"Het was wel lekker stevig inderdaad. Je had wel het gevoel dat je steun had. (…) Nee [de brace is nooit beschadigd geraakt of kapotgegaan terwijl ik hem gebruikte]."<br>*It was quite solid indeed. It felt like it was giving support. No, the brace has never been damaged or broken while using it.*<br>(F, 75, KL 3, 4 weeks of intermittent brace use)	
		"In het begin was [de stevigheid] wel even een beetje wennen, maar als je er een beetje aan gewend bent, (…) Nee [er is nooit iets kapot gegaan] (…) Eén keer was er zo'n dopje afgevallen, maar die kon je er zo weer in klikken."<br>*At first the brace's sturdiness took a bit of getting used to, but once you get used to it…. No, nothing broke. One time this little cap came off, but you could just click that back in.*<br>(M, 56, KL 2, 24 weeks of brace use)	
		"Ik had hem steviger verwacht [maar] deze heeft vooralsnog voor mij hetgeen gedaan waarvoor hij is. (…) Nee [er zijn geen dingen kapot gegaan en ik heb er geen problemen mee gehad]."<br>*"I expected it to be sturdier, but so far this one has done for me what it was supposed to. No, nothing has broken and I haven't had any problems with it.*<br>(F, 55, KL 2, 24 weeks of brace use)	
		"[De stevigheid van de brace was] Heel goed. Ja, zeker. (…) Nee, niets [beschadigd of kapot geweest]."<br>*"The sturdiness of the brace was very good. Yes, definitely. No, nothing was damaged or broken.*<br>(F, 59, KL 3, 8 weeks of brace use)	
		"[De stabiliteit van de brace] vind ik fantastisch. Aan alle kanten waar je hem hebt, zit stevigheid. (…) Nee [de brace is tijdens het gebruik nooit kapot geweest of beschadigd geraakt]."<br>*I find the stability of the brace fantastic. It provides stability all around. No, the brace has never been broken or damaged during use.*<br>(M, 75, KL 2, 23 weeks of brace use)	

		"Ik vond hem redelijk stevig. (…) Nee, nooit [beschadigd geraakt of kapot gegaan]."<br>*"I found it reasonably solid. No, it never got damaged or broken.*<br>(M, 52, KL 3, unknown number of weeks of brace use)	
		"Een stevig ding is het. Laatst had ik hem om. Toen was ik bij mijn broer even bezig. Toen bleef ik met mijn voet achter een pallet haken. De bril heeft meer geleden dan mijn knie [en] mijn brace. (…) Nee [de brace is niet beschadigd geraakt, kapot geweest en ik heb er geen problemen mee gehad]."<br>*It's a strong thing. I was wearing it the other day when I was at my brother's place. Then my foot got caught behind a wooden pallet. My glasses suffered more than my knee and my brace. No, the brace didn't get damaged, it wasn't broken, and I didn't have any problems with it.*<br>(M, 60, KL 2, 24 weeks of brace use)	
	Weight	"Ik merk hem niet."<br>*I don't notice it.*<br>(M, 60, KL 3, 24 weeks of brace use)	"Ik vind hem behoorlijk zwaar."<br>*I find the brace quite heavy*<br>(F, 75, KL 3, 4 weeks of intermittent brace use)
		"[Het gewicht] is geen probleem geweest. (…) Daar heb ik geen moeite mee gehad, niets. (…) Ik had niet het gevoel, ik moet iets meeslepen of zo. Hij zit en het is goed."<br>*The weight hasn't been a problem. I haven't had any trouble with that, nothing. I didn't feel like I had to drag something along or anything. It's there, and it's fine.*<br>(F, 67, KL 3, 24 weeks of brace use)	"Nou, ik vond het wel zwaar aan mijn been. Soms sleepte ik met mijn been. Dan denk ik: 'oeh, wat heb ik toch een gewicht aan mijn been hangen.'"<br><br>*Well, I found it to be quite a weight on my leg. Sometimes I was dragging my leg. Then I thought 'my, what a weight on my leg that is.'*<br>(F, 59, KL 3, 8 weeks of brace use)
		"Ja, daar ben ik aan gewend. Het is niet voor mij te zwaar."<br>*Yeah, I'm used to it. For me it is not too heavy.*<br>(F, 74, KL 2, 22 weeks of intermittent brace use)	
		"[Het gewicht daar had ik] Geen last van."<br>*The weight didn't bother me.*<br>(M, 56, KL 2, 24 weeks of brace use)	
		"Dat is ook prima. Het is geen zwaar ding, dat valt reuze mee. Ik heb niet de hele dag het idee van, ik heb een brace om. Dat heb ik niet, nee."<br>*The weight was fine too. It's not a heavy thing, it was allright really. I didn't have that feeling of 'I'm wearing a brace' all day. No, I didn't.*<br>(F, 55, KL 2, 24 weeks of brace use)	
		"[Het gewicht van de brace was] Prima. Niet te zwaar."<br>*The weight of the brace was fine. Not too heavy.*<br>(M, 75, KL 2, 23 weeks of brace use)	
		"Nee, hij was vrij licht, dus echt zwaar was die niet."<br>*No, it was fairly light, so it wasn't heavy, really.*	

		(M, 52, KL 3, unknown number of weeks of brace use)	
		"[Over het gewicht van de brace ben ik] Zeer tevreden. Je merkt het niet echt. Je merkt alleen als je hem er omheen doet en aantrekt. Dan denk je 'o, ja, er zit wat omheen.' Maar voor de rest, nee, ik loop er niet langzamer door. Ik ben sneller geworden." *I'm very satisfied about the weight of the brace. You don't really notice it. You only notice it when you put it on and strap it in. Then you think, 'oh yeah right, there's something there.' But otherwise, no, it doesn't slow me down. I've actually become faster.* (M, 60, KL 2, 24 weeks of brace use)	
	Safety	"Nee [de brace heeft nooit gevaarlijke situaties opgeleverd]." *No, the brace has never caused any dangerous situations.* (F, 67, KL 3, 24 weeks of brace use)	
		"Nee [ik ben nooit door de brace heen gezakt en hij is nooit uit zijn slot geschoten]." *No, I've never buckled through the brace and the locking system never gave way.* (M, 56, KL 2, 24 weeks of brace use)	
		"Nee [ik ben nooit door de brace heen gezakt]." *No, I've never buckled through the brace.* (F, 55, KL 2, 24 weeks of brace use)	
		"Nee [ik ben nooit door de brace heen gezakt]. Het voelde gewoon heel stevig, (…)." *No, I've never buckled through the brace. It just felt very solid.* (F, 59, KL 3, 8 weeks of brace use)	
		"Nee [ik ben nooit door de brace heen gezakt]." *No, I've never buckled through the brace.* (M, 75, KL 2, 23 weeks of brace use)	
		"[Ik had nooit het idee dat de brace] stuk zou kunnen gaan, of dat ik er doorheen zou kunnen gaan. Ik had wel het vertrouwen erin dat hij alles hield." *It never felt like the brace could break or that I could buckle through it. I was confident that it would hold everything.* (M, 52, KL 3, unknown number of weeks of brace use)	
		"Nee [ik ben nooit door de brace heen gezakt]." *No, I've never buckled through the brace.* (M, 60, KL 2, 24 weeks of brace use)	

Supporting Table 1E. Participants' concluding perspectives about their overall experiences and satisfaction with the brace.

Concluding perspectives about (using) the brace	"Ik had gedacht dat als je 4, 5 dagen de brace om had dat de pijn weg was. Dat je dan even zonder brace weer een paar dagen pijnloos kon lopen, maar dat is het niet. (…) Dat effect heeft het niet gegeven. (…) Ja, ik denk als ik er hinder van heb en ik kan de knie ontlasten, dan zal ik [de brace in de toekomst] best wel aandoen. (…) Als je de brace aanhebt, worden [de klachten] wel een stuk minder, (…) maar zo gauw als je [de brace] af hebt, is [de klacht] weer terug. (…) Het ondersteunde wel, maar het werd niet beter. Het is eigenlijk bergafwaarts gegaan. (…) Ik heb er jammer genoeg niet veel baat bij gehad. (…) Het zal niet aan de brace liggen, maar het ligt aan de knie."<br>*I had thought that once I'd wear the brace for 4, 5 days that the pain would be gone. And that I could then walk pain-free without the brace for a couple of days. It didn't have that effect. I think if my knee bothers me and the brace can relieve some of the load on my knee, then I will probably wear the brace in the future. Wearing the brace does ease my symptoms, but once I take it off the symptoms are back. The brace provided support, but it didn't make it better. It actually went downhill. Unfortunately, I didn't benefit much from it. It probably isn't because of the brace, but it's my knee.*<br>(M, 60, KL 3, 24 weeks of brace use)
	"De brace op zich is volgens mij een hele goede uitvinding, maar ik heb er eigenlijk niet het voordeel van wat ik gehoopt had. (…) Ik denk [dat ik 'm wel blijf ga dragen] zolang er geen andere oplossing is."<br>*I think the brace is a very good invention, but I haven't actually had the benefit that I had hoped for. I think I will keep wearing it as long as there is no other solution.*<br>(F, 67, KL 3, 24 weeks of brace use)
	"Ik denk (…) niet [dat mijn been rechter staat]. [Over het algemeen zou ik mijn tevredenheid uitdrukken met] toch zeker wel een [cijfer] acht (…) want hij is zeker een acht waard qua lopen. (…) [Ik ga de brace in de toekomst nog blijven dragen], zeker als we echt gaan wandelen en als ik denk ik naar de stad ga. (…) [De invloed op mijn klachten kwam misschien van] de [pijn] medicijnen en de brace samen."<br>*I don't think I've had that correction of my lower leg. Overall I would grade my satisfaction with an eight out of ten because it is definitely worth an eight in terms of walking. I will continue to wear the brace in the future, especially when we're going for a real walk or if I plan to go into town. The positive influence on my symptoms may have resulted from the pain medication and the brace combined.*<br>(F, 74, KL 2, 22 weeks of intermittent brace use)
	"Ik heb hem anderhalve maand gehad of zo. Ik vond dat echt te veel. Dat is wel een beetje demotiverend. Als er te veel dingen tegenwerken, dat je dan inderdaad denkt 'ja, klaar.' (…) Eigenlijk ben ik dus niet zo tevreden. De nadelen waren groter dan de voordelen. De voordelen die merk je niet op korte termijn, maar de nadelen merk je gelijk. (…) [Wat de meeste invloed had op mijn ontevredenheid is] dat je hem niet makkelijk zelf aandoet. (…) Nee. Ik hoop dat het niet nodig is [om de brace in de toekomst nog te dragen] (…) Ik [krijg een totale knie arthroplastiek] en dan moet het hopelijk beter gaan."<br>*I've used the brace for about a month and a half. I found that to be too much. That's a bit demotivating. When too many things work against you, you indeed think 'yeah, I'm done with it.' So I'm not that satisfied actually. The disadvantages were greater than the benefits. You don't notice the benefits in the short term, but you immediately notice the disadvantages. My dissatisfaction is mostly influenced by the fact that it wasn't easy to put the brace on myself. No. I hope it won't be necessary to wear the brace in the future. I'm having a total knee replacement and hopefully that wil improve things.*<br>(F, 75, KL 3, 4 weeks of intermittent brace use)
	"Ja, het heeft me wel geholpen. (…) Ik denk dat ik hem [in de toekomst] wel ga gebruiken, ja. Vooral op het werk gebruik ik hem altijd, omdat ik daar de meeste belasting [op mijn knie] heb. (…) Dat [de brace de knie] naar binnen drukte en dat ik meer speling krijg hier aan de zijkant dat hielp goed."<br>*Yes, the brace did help me. I think I will continue to use it in the future, yes. Especially at work I always use it because that's when I have most of the physical strains on my knee. The fact that the brace pushed my knee inward and that I got more space here on this side that helped a lot.*<br>(M, 56, KL 2, 24 weeks of brace use)
	"Hij voelt niet zwaar, hij zit niet irritant, dus dat zijn allemaal wel dingen die meespelen. (…) Ik heb er eigenlijk alleen maar gemak van. (…) Ja [ik ga de brace in de toekomst nog dragen]. (…) met sporten, of we zeggen we gaan een heel eind lopen dat ik hem toch wel om ga doen ter voorkoming van."

	It doesn't feel heavy, it's not annoying, so these are all factors that play a role. I actually only experienc benefits from it. Yes, I will wear the brace in the future, for example with sports or when we're going for a long walk, just as a kind of preventive measure.<br>(F, 55, KL 2, 24 weeks of brace use)
	"Op een gegeven had ik 's avonds meer last van mijn knie als ik hem afdeed dan (…) als ik hem omhad. (…) Ik had er eigenlijk meer last van dan gemak. (…) Ik heb er eigenlijk heel eerlijk niet veel van gemerkt."<br>*At one point my knee bothered me more when I took the brace off compared to when I was wearing it. It actually bothered me more than it was convenient. In all honesty, I didn't really notice that much of it.*<br>(M, 51, KL 2, 1 week of brace use)
	"[De brace had] niet het effect wat ik verwacht had. (…) Ik had verwacht dat de pijn af zou nemen. De slijtage zit aan de binnenkant van mijn knie, dus ik dacht dat dat drukken op de buitenkant van mijn knie de pijn zou verminderen. (…) Misschien is mijn verwachting te hoog. (…) Misschien [ga ik de brace in de toekomst] toch wel [dragen] op mijn werk. (…) Misschien dat ik toch zeg 'laten we eens kijken of ik het verschil weer merk.' (…) Op mijn vrije dagen thuis had ik hem nooit om, alleen op mijn werk. Daardoor kon ik goed het verschil beoordelen, en merkte ik geen verschil of ik hem nou wel of niet omhad."<br>*The brace didn't have the effects that I expected. I expected the pain to decrease. The wear and tear is on the inside of my knee so I thought that putting pressure on the outside of my knee would reduce the pain. Maybe my expectations were too high. Maybe I'll wear the brace at work in the future. Maybe I'll say 'let's see if I notice the difference again.' I never wore it during my days off when I was at home, only at work. That really allowed me to evaluate the difference, and I didn't notice any difference whether I wore it or not.*<br>(F, 59, KL 3, 8 weeks of brace use)
	"Ik had wel iets kromme benen, maar [dat is niet erger geworden]. (…) Ik ben er tevreden mee. Zijn werk doet hij. (…) Ja [ik ga de brace in de toekomst blijven dragen want] Ik denk dat ik er ook veel baat bij heb."<br>*I was slighty bow-legged, but that hasn't worsened. I'm satisfied with the brace. It's doing its job. Yes, I will continue to wear the brace in the future because I think I benefit from it a lot.*<br>(M, 75, KL 2, 23 weeks of brace use)
	"Nee [mijn verwachtingen] zijn niet uitgekomen [, maar dat] lag ook deels aan het feit dat mijn knie zodanig slecht is dat de enige optie nog een nieuwe knie is. (…) Dat hij licht is en een blits uiterlijk [heeft, heeft de meeste invloed op mijn tevredenheid]. (…) Nee, [ik zou de brace in de toekomst] helemaal niet meer [aan doen]. (…) [De invloed van de brace was dat ik] meer knieklachten [kreeg]. (…) [Dat uitte zich] wanneer ik de brace eigenlijk weer afdeed (…) Het was meer dat ik het als vervelend heb ervaren, dan als dat ik dacht van 'Oh, dat is goed.'"<br>*No, my expectations were not met, but that was partly due to my knee being so bad that the only option left is a knee replacement. The fact that the brace is light and has a flashy appearance influenced my satisfaction the most. No, I'm not going to wear the brace in the future anymore. The influence of the brace was that I got more knee complaints. This manifested itself through an increase in symptoms each time I took the brace off. Overall, I thought the brace was more annoying than thinking 'Oh, that's good'*<br>(M, 52, KL 3, unknown number of weeks of brace use)
	"Ja [mijn verwachtingen zijn uitgekomen]. Zo gauw hij er omheen zit [heb ik] minder pijn. (…) Het is stevig. (…) Ik vind hem fijn lopen. (…) Het is een soort dwangbuis om de knie. (…) Als hij er omheen zit, heb ik er gemak van. (…) Doe ik het niet, heb ik er mijn eigen mee. (…) Het heeft gebaat, maar het heeft het probleem niet weggehaald (…) Ja [ik ga de brace blijven dragen de komende tijd]. Ik weet niet wanneer ik me nou weer bij de orthopeed moet melden voor, hoe nou verder. Als hij zegt van 'ik zet er nieuwe [knie] in', oké, dan bewaar ik hem want misschien kan ik hem overhouden voor rechts."<br>*Yes, my expectations were met. As soon as I have the brace on I have less pain. It's sturdy. I find it comfortable to walk with. It's like a straightjacket for the knee. When it's on I can feel the benefits. If I don't wear it, I'm just shooting myself in the foot. It has helped, but it hasn't solved the problem. Yes, I will continue wearing the brace in the time to come. I don't know when I should see the orthopaedic surgeon again to see what needs to be done next. If he says 'I'll put in a new knee', then I'll just keep the brace because maybe I can save it for my other knee.*<br>(M, 60, KL 2, 24 weeks of brace use)
