# Supplementary material for: Effectiveness and user experiences of a valgus brace in patients with knee osteoarthritis: A mixed-method randomised controlled trial
Source: PLoS One. 2025 Sep 4;20(9):e0330157. doi: 10.1371/journal.pone.0330157 (PMC12410808; doi:10.1371/journal.pone.0330157)
Supplement: S2 Table — (PDF) [file pone.0330157.s006.pdf]

S2 Table. Triangulation of quantitative outcomes and qualitative (sub)themes in overlapping areas of data.

Quantitative outcome	Qualitative (sub)theme[a]	Triangulation, interpretation and discussion
**Knee pain**	**Body function: Pain**	
• No statistically significant difference between groups in VAS scores for pain at rest.	• Approximately half the participants perceived less pain, while others noted no change, short-lived relief, or increased pain.	• Findings varied across participants, with some reporting benefit and others not.
• Strong statistically significant and clinically relevant difference between groups in VAS scores for pain intensity after a 6-MWT; the brace users had less pain.	• Participants with more severe OA perceived the brace as less effective for pain relief.	• Averaging VAS pain data across all brace users may have obscured individual variations in treatment response.
• Brace users ingested significantly lower amounts of analgesics than control participants.		• Concurrent analgesic use during the bracing period may have influenced pain score outcomes. Resulting sample sizes were too small for a robust sensitivity analysis.
**Knee physical functioning**	**Body function: Muscle functions**<br>**Activities: Walking and moving**	
• No statistically significant difference between groups in 6-MWT and WOMAC scores.	• The majority of participants perceived improved knee stability and movement control, with minimal reports of muscle weakness. Two participants reported signs of leg muscle atrophy.	• Findings varied across participants, with some reporting benefit and others not. While the brace did not impact 6-MWT and WOMAC scores, qualitative responses suggest perceived improvements in muscle function for many brace users.
	• Several participants felt that using the brace had positively influenced their stair walking, lifting and carrying objects and moving around using transportation.	• Averaging WOMAC and 6-MWT data across all brace users may have obscured individual variations in treatment response.
	• Some participants complained that the brace was a hindrance during kneeling or using transportation, others said the brace had not or only somewhat restricted their overall knee movements.	• The 6-MWT may lack sensitivity to detect subtle changes in knee physical functioning following brace use.

Quantitative outcome	Qualitative (sub)theme[a]	Triangulation, interpretation and discussion
**Generic health status**	**Body function: Emotional functions (b152)**	
• No statistically significant difference between groups in SF-12 scores.	• Most participants reported not to have had feelings of shame related to brace use.<br><br>• One participant reported an increased confidence in their knee.	• Given the minimal conceptual overlap between the SF-12 and the qualitative data, triangulation was not deemed appropriate.<br>• Qualitative data aligned between brace users regarding feelings of shame as a (ICF) component of emotional functions
**Physical complications**	**Body function: Structure of areas of skin (s810)**	
• The proportion of brace users reporting skin-related complications dropped from 57% to 17% in the first three weeks, with occasional reports thereafter.	• Participants experiencing one or more skin-related complications suggested that they were not overly bothered by these.	• Findings were consistent across participants. Skin-related complications were transient and appeared associated with the break-in phase.
**Brace fit and satisfaction**	**Environmental factors: Assistive products and technology for personal use in daily living (e1151)**	
• The proportion of brace users reporting fit issues decreased from 60% to 40% over two weeks, with occasional minor issues reported thereafter.<br>• VAS satisfaction scores increased by 10 mm (clinically relevant) but the change was not statistically significant.	• While some participants reported some minor issues relating to the brace's fit, the majority of participants said the brace fitted well.<br>• Most participants expressed satisfaction with the brace.	• Findings regarding the brace's fit were consistent across participants. Most issues appeared to be associated with the break-in period.<br>• Findings converged in showing improved satisfaction. While satisfaction increased meaningfully for many participants, this change was not reflected in VAS scores, possibly due to limited sensitivity of the scale or small sample size.
**Severity of knee osteoarthritis**	**Body function: pain**<br>**Activities: Walking and moving**	
• A higher proportion of participants with Kellgren & Lawrence grade 3[b] from the intervention group (58%) underwent knee arthroplasty surgery compared to the control group (27%) and all interviewed participants with grade 3 had surgery. Surgery rates among participants with grade 2 were low and similar between groups.	• Participants with more severe OA perceived the brace as less effective in terms of reducing knee pain and perceived more activity limitations during kneeling or moving around using transportation.	• Findings converged in suggesting that the brace was less effective for participants with more severe OA, potentially prompting them or the orthopaedic surgeon to opt for arthroplasty surgery.

[a] Qualitative (sub)theme terminology and coding is derived from the World Health Organization's *International Classification of Functioning, Disability and Health* (ICF), version 2025-01. [b]The Kellgren & Lawrence classification system grades the severity of knee osteoarthritis based on radiographic findings. Patients with grade 2 have *"Definite osteophytes and possible joint space narrowing"*, patients with grade 3 have *"Moderate multiple osteophytes, definite joint space narrowing, some sclerosis (increased bone density), and possible deformity of the bone contours"*.

6-MWT, 6-Minute Walk Test; ICF, International Classification of Functioning, Disability and Health; OA, osteoarthritis; SF-12, Short Form-12; VAS, Visual Analogue Scale; WOMAC, Western Ontario and McMaster Universities Osteoarthritis Index.
