# Supplementary material for: Effectiveness and user experiences of a valgus brace in patients with knee osteoarthritis: A mixed-method randomised controlled trial
Source: PLoS One. 2025 Sep 4;20(9):e0330157. doi: 10.1371/journal.pone.0330157 (PMC12410808; doi:10.1371/journal.pone.0330157)
Supplement: Attachment — Submitted filename: comment.docx [file pone.0330157.s007.docx]

I have carefully reviewed the manuscript titled “Effectiveness and user experiences of a valgus brace in patients with knee osteoarthritis: A mixed-method randomised controlled trial.”

While this is an important and valuable contribution, several methodological, reporting, and presentation issues need to be addressed before the manuscript can be considered for publication.

**Below are my detailed comments:**

**Major Issues**

Abstract Reporting:

The effect size of the main outcome should be explicitly reported in the Abstract alongside the p-values.

The number of participants per group (n=23) should be stated in the Abstract Methods section, not in the Results.

Effect Size Interpretation in Main Text:

Beyond statistical significance, the authors should interpret and discuss the clinical magnitude of the effects (e.g., Cohen’s d or appropriate effect size) in the Discussion section.

Insufficient Description of the Brace:

Although the SecuTec® OA brace is mentioned, there is no clear orthosis and technical description of its biomechanical mechanism.

Such details are essential for replication and clinical relevance.

Reference Needed for Literature Gap:

Line 87: The claim about the lack of qualitative studies evaluating orthotic and assistive devices requires a supporting reference.

Wide Age Range Needs Acknowledgment:

Line 100–102: Including participants aged 40–75 introduces potential heterogeneity. This should be acknowledged explicitly as a limitation.

[NEW] Limited Generalizability:

The study’s findings may have limited generalizability due to the wide age range (40–75 years), varying BMI profiles (with an exclusion criterion of BMI >35), and potential differences in disease severity or comorbidities among participants. This aspect should be critically addressed as a limitation to provide a more balanced interpretation of the results.

Reference Needed for BMI Cutoff:

Line 110: A reference supporting the exclusion criterion of BMI >35 is needed to justify its clinical relevance.

Control Group Design Concerns:

Line 173–177: Allowing the control group to pursue any standard treatment (physiotherapy, injections) except bracing may introduce significant variability and confounding. This needs to be critically discussed as a methodological limitation.

Tables Formatting and Completeness:

Baseline Characteristics Table (Table 2): Should report p-values to confirm baseline comparability between groups.

Units of Measurement: All variables should have clearly stated units (e.g., meters, degrees, cm).

Footnotes: Should provide expanded explanations regarding missing data.

Consistency: Data formats (mean (SD), n (%)) should be standardized across all tables.

Adjusted Analyses: Table 3 (co-interventions) should ideally include or at least discuss adjustment analyses for potential confounders.

Figures Quality and Completeness:

Figures, especially the CONSORT diagram, should be provided at higher resolution (minimum 300 dpi) to ensure clarity.

Figure legends should be expanded to be fully self-explanatory.

In-text references to figures should be clearer.

Inclusion of a schematic illustration or technical diagram of the valgus brace’s mechanism would greatly improve readers’ understanding.

Sample Size and Power Issues:

The final analyzed sample size (n=46) was notably lower than planned, leading to an underpowered study. The risk of Type II error should be more strongly emphasized.

Randomization Before Eligibility Confirmation:

Line 124–149: Randomizing participants before full eligibility confirmation led to post-randomization exclusions, which can introduce selection bias.

Lack of Blinding:

Line 127: Neither participants nor assessors were blinded, increasing the risk of measurement bias, particularly for subjective outcomes like VAS.

The language is professional and clear, with only minor grammatical improvements needed.

Summary Recommendation

The manuscript addresses a clinically important question with a valuable mixed-methods design.

However, substantial revisions are needed to address issues regarding outcome measurement appropriateness, complete and transparent reporting (especially in tables and figures), stronger methodological justifications, and clearer acknowledgment of study limitations.

Addressing these concerns will significantly enhance the manuscript’s scientific rigor, transparency, and potential clinical impact.

Discussion Section Suggestions:

In the Discussion section, the authors should expand on the limitations identified, including the potential impact of the wide age range (40–75 years), varying BMI profiles (despite the exclusion of BMI >35), and other participant characteristics (e.g., disease severity or comorbidities). These factors may limit the generalizability of the findings to broader populations or different clinical settings. The authors should discuss how these heterogeneities might influence the applicability of the results and suggest directions for future research, such as conducting subgroup analyses or targeting more homogeneous cohorts to validate the findings.
