# Supplementary material for: Effectiveness and user experiences of a valgus brace in patients with knee osteoarthritis: A mixed-method randomised controlled trial
Source: PLoS One. 2025 Sep 4;20(9):e0330157. doi: 10.1371/journal.pone.0330157 (PMC12410808; doi:10.1371/journal.pone.0330157)
Supplement: Attachment — Submitted filename: Response to Reviewers.docx [file pone.0330157.s009.docx]

**Response to Reviewers**

PONE-D-25-04724 (PLOS ONE)
Effectiveness and user experiences of a valgus brace in patients with knee osteoarthritis:

a mixed-method randomised controlled trial.

*Editorial comments* and subsequent reply and/or amendment

*“1. Please ensure that your manuscript meets PLOS ONE's style requirements, including those for file naming.”*

The manuscript was prepared using the PLOS ONE style requirement document and as far as we are aware it meets all the requirements listed there.

*“2. Thank you for uploading your study's underlying data set. Unfortunately, the repository you have noted in your Data Availability statement does not qualify as an acceptable data repository according to PLOS's standards. At this time, please upload the minimal data set necessary to replicate your study's findings to a stable, public repository (such as figshare or Dryad) and provide us with the relevant URLs, DOIs, or accession numbers that may be used to access these data. For a list of recommended repositories and additional information on PLOS standards for data deposition, please see* [*https://journals.plos.org/plosone/s/recommended-repositories*](https://journals.plos.org/plosone/s/recommended-repositories)*.”*

We have moved the datasets to figshare. We have also added a new file there in response to comment 5 of reviewer #1. Here you can find private links to these three files:

- S1 File (<https://figshare.com/s/50cacee63c01e2c2f8a6>) on page 19, line 321 and page 20, line 351.

- S1 Table (<https://figshare.com/s/20212f8bb7fba462d17f>) on page 23, line 395.

- S2 Table (<https://figshare.com/s/7c3894a86e4b00872d41> on page 23, line 398.

Upon final acceptance of the manuscript we will insert the definitive DOIs.

*Comments Reviewer #1* and subsequent reply and/or amendment

*“1. The study was underpowered due to an insufficient sample size (n=23 per group) compared to the initial target of 80 participants, increasing the likelihood of Type II errors, and the sample size calculation does not appear to account for potential attrition rates beyond a general 15% estimate.* *The authors should clearly state how the final sample size impacts the interpretation of non-significant findings and conduct a post hoc power analysis to determine if the study was sufficiently powered to detect meaningful differences in secondary outcomes.”*

We agree that the study was underpowered to detect between-group differences, not only in the secondary outcomes but in nearly all outcomes overall. To clarify this, we have revised two sentences in the Discussion (page 36, lines 707-710). We hope this, along with the acknowledgements in the first paragraph of the Discussion (page 31, lines 602–603) and in the Abstract’s Conclusion section, now clearly communicates this limitation to the reader. Regarding post hoc power analysis, we have not included one, as it is mathematically redundant in the context of non-significant findings and would simply reiterate the lack of statistical power already discussed.

*“2. The manuscript does not provide a clear strategy for handling missing data beyond simple mean imputation for some questionnaire-based measures (e.g., SF-12 and WOMAC), and it is unclear whether missing data were missing completely at random (MCAR), missing at random (MAR), or missing not at random (MNAR). I would recommend using multiple imputation or sensitivity analyses to assess the impact of missing data on the robustness of results and report the extent of missing data for each outcome measure while justifying the chosen imputation method.”*

The missing data in our study occurred primarily due to COVID-19-related social restrictions that prevented in-person assessments during a specific period. As this missingness was unrelated to participant characteristics or outcomes, we consider it to be Missing Completely at Random (MCAR). We have added wording to the limitations sections of the Discussion (page 36, line 712-714) to clarify this.

Experts have indicated that in cases of MCAR, complete-case analysis does not introduce bias, and multiple imputation is not necessary. Moreover, given the relatively small sample size (23 per group), we would be hesitant to apply multiple imputation, as it may introduce instability in estimates due to limited information available for imputation. We have therefore chosen not to apply imputation methods in this context.

*“3. Multiple statistical tests were performed, yet no correction for multiple comparisons (e.g., Bonferroni or Benjamini-Hochberg) is mentioned, increasing the risk of Type I errors. The authors should apply an appropriate correction method for multiple comparisons or justify why it was not deemed necessary and consider reporting effect sizes alongside p-values to provide a clearer clinical interpretation.”*

We appreciate the reviewer’s suggestion to adjust for multiple comparisons. However, we have chosen not to apply a Bonferroni or similar correction for several reasons grounded in both statistical and conceptual considerations:

1. The outcomes assessed in this study—while all related to patient functioning—represent the distinct clinical constructs of pain (at rest and after walking), knee functioning (6-MWT, WOMAC) and generic health status (SF-12). These are not independent or interchangeable endpoints, but rather complementary dimensions of the patient experience. Consequently, we view our analysis not as a set of isolated hypothesis tests, but as an effort to characterize a multifaceted outcome.

2. Our outcomes were pre-specified and based on clinically relevant constructs, rather than selected post-hoc based on significance. As such, we are not conducting exploratory analyses that would require strong control of the family-wise error rate. In line with current recommendations in the statistical literature, we prioritise transparency by reporting all effect estimates with accompanying 95% confidence intervals, which allow readers to evaluate both the magnitude and the uncertainty of observed effects.

3. Only one pain-related outcome showed significant between-group differences. Therefore, applying a Bonferroni correction would not meaningfully alter the overall pattern of results. In fact, it would increase the risk of overlooking potentially meaningful effects (i.e., increase the risk of a type II error).

For the reasons mentioned above, and in line with current best practices in clinical and observational research, we have chosen to retain our current presentation of results without *p-*value adjustment, while ensuring all findings are interpreted with appropriate caution.

*“4. While multilevel modeling is an appropriate choice, the manuscript does not provide details on why a random intercept model was preferred over alternatives such as random slopes or mixed-effects models with covariate adjustments, and it is unclear whether model assumptions (e.g., normality of residuals, homoscedasticity) were tested. I would recommend that the authors should provide diagnostic checks (e.g., residual plots, normality tests) to confirm model appropriateness and consider alternative models, such as a repeated-measures ANCOVA adjusting for baseline covariates.”*

We have rewritten the entire ‘Quantitative data collection and analysis’ section on page 14 and provided additional information about testing the model assumptions here (line 253-261) and in the ‘Primary outcome’ results section on page 20 (line 347-350).

*“5. The qualitative findings suggest benefits not detected in quantitative analysis, raising concerns about potential bias in either data collection or interpretation, and no integration framework (e.g., triangulation analysis) is used to reconcile these discrepancies. I recommend that the authors consider using a joint display table to directly compare quantitative and qualitative findings and discuss how selection bias in the qualitative sample might have influenced results.**”*

We agree with the reviewer that a comprehensive table, triangulating the quantitative outcomes and qualitative (sub)themes in overlapping areas of data, is indeed useful.

Because this new table is rather large, we have decided to add it as a Supporting Information (S2) Table. We have tentatively added an in-text reference to the S2 Table on page 23 (line 395-398) and added a private link to the file on figshare (please see <https://figshare.com/s/7c3894a86e4b00872d41>). We will insert an activated accompanying DOI into the manuscript upon final acceptance.

If the editor or reviewer feel this table is best added to the manuscript as a regular table, we are also happy to add it there (as Table 4). Please advise.

While we appreciate the reviewer’s concern regarding potential selection bias in the qualitative sample, we found no indication that the characteristics of the interview subsample differed substantially from those of the parent sample.

*“6.* *Although co-interventions (e.g., analgesic use, physiotherapy) were recorded, no clear sensitivity analyses were conducted to assess their impact on primary outcomes. Can authors conduct subgroup or sensitivity analyses to explore whether co-interventions influenced the observed effects and report whether these factors were included as covariates in the regression models?”*

Please note that on page 33 and 34 (line 647-650) we already explained that we refrained from performing a sensitivity analysis of co-interventions because the resulting sample sizes would be too small to draw any meaningful conclusions.

*“7. The study only evaluates outcomes up to six months, which may not be sufficient to assess long-term efficacy and adherence to brace use. The Authors should discuss the need for follow-up studies examining long-term effects beyond six months and consider analyzing long-term adherence and its relationship with effectiveness.”*

We agree with the reviewer that investigating six months of brace use may not be sufficient to assess long-term efficacy and adherence to brace use. This is exactly why we already recommended conducting well-powered and high-quality studies, with follow-up assessments beyond 6 months of brace use, to improve evaluations of the effectiveness of individually fitted knee braces on body functions, activities, participation and wear time (on page 36, line 730-737).

*Comments Reviewer #2* and subsequent reply and/or amendment

*“The effect size of the main outcome should be explicitly reported in the Abstract alongside the p-values.”*

*&*

*“Beyond statistical significance, the authors should interpret and discuss the clinical magnitude of the effects (e.g., Cohen’s d or appropriate effect size) in the Discussion section.”*

We appreciate the reviewers’ interest in effect size metrics. However, we respectfully suggest that adding a standardized effect size such as Cohen’s *d* is not the most appropriate or informative approach in the context of our analyses.

Our study employs multilevel linear regression models on longitudinal data, capturing both within-person changes over time and between-group differences. We have deliberately chosen to report unstandardized regression coefficients along with their 95% confidence intervals, as this provides a direct, interpretable, and contextually grounded estimate of the effect magnitude and its uncertainty. This approach is particularly important when the outcome measure has intrinsic meaning (e.g., a clinical or validated scale), allowing readers to evaluate the practical significance of changes without the obfuscation of unitless transformations.

While standardised effect sizes like Cohen’s *d* are often used in more simplistic analyses (e.g., t-tests), their application in multilevel models—particularly with repeated measures—is methodologically fraught. There is no single agreed-upon method for calculating *d* in these contexts, as it depends on whether variance is pooled across individuals, timepoints, levels of the model, or residuals. Consequently, standardized effect sizes in multilevel frameworks often obscure rather than clarify the findings, and risk misinterpretation if readers are unaware of the underlying calculation choices.

We believe that the model-based estimates with confidence intervals presented in our manuscript provide a richer and more accurate depiction of the intervention’s effect than a single scalar like Cohen’s *d*. We strongly feel that introducing *d* would not enhance interpretability, but instead reduce the clarity and specificity of the findings we report. Nevertheless, if the editorial team requires a standardised effect size for consistency with journal guidelines, we are willing to include it in the existing S1 File—alongside a brief explanation of its limitations in the current context. However, we respectfully emphasize that the unstandardised model estimates remain the most meaningful and scientifically valid representation of our results.

*“The number of participants per group (n=23) should be stated in the Abstract Methods section, not in the Results.”*

Amended as suggested (on page 2, line 27 and 29).

*“Although the SecuTec® OA brace is mentioned, there is no clear orthosis and technical description of its biomechanical mechanism. Such details are essential for replication and clinical relevance.”*

&

*“Inclusion of a schematic illustration or technical diagram of the valgus brace’s mechanism would greatly improve readers’ understanding.”*

We have added a URL linking to the manufacturer’s international webpage for the specific brace that was used during the study (page 8, lines 157–159).

*“Line 87: The claim about the lack of qualitative studies evaluating orthotic and assistive devices requires a supporting reference.”*

We agree that claims about a lack of research should be supported. However, our assertion is based precisely on the absence of qualitative studies in this area. We have slightly tweaked the sentence (page 5, line 87) to address this suggestion.

*“Line 100–102: Including participants aged 40–75 introduces potential heterogeneity. This should be acknowledged explicitly as a limitation.”*

*&*

*“The study’s findings may have limited generalizability due to the wide age range (40–75 years), varying BMI profiles (with an exclusion criterion of BMI >35), and potential differences in disease severity or comorbidities among participants. This aspect should be critically addressed as a limitation to provide a more balanced interpretation of the results.”*

*&*

*“In the Discussion section, the authors should expand on the limitations identified, including the potential impact of the wide age range (40–75 years), varying BMI profiles (despite the exclusion of BMI >35), and other participant characteristics (e.g., disease severity or comorbidities).* *These factors may limit the generalizability of the findings to broader populations or different clinical settings. The authors should discuss how these heterogeneities might influence the applicability of the results and suggest directions for future research, such as conducting subgroup analyses or targeting more homogeneous cohorts to validate the findings.”*

Here we combine our response to these three comments, as we feel they were similar in nature.

We have added a sentence to the limitations section of the Discussion (page 37, line 722-726) to acknowledge these limitations. Additionally, in the future research section of the Discussion (page 37, line 735) we have added wording to indicate that future research on the influence of age and physical activity is (also) warranted.

*“Line 110: A reference supporting the exclusion criterion of BMI >35 is needed to justify its clinical relevance.”*

We only included patients with a BMI ≤ 35 because patients with BMI values *>*35 could not be fitted with the type of brace we used. As such, this inclusion (or rather exlusion) criterion was more of a pragmatic choice. We were unable to find individual studies that were supporting this cutoff BMI value. If the reviewer is aware of any previous studies providing such a reference, we would be happy to cite it.

*“Line 173–177: Allowing the control group to pursue any standard treatment (physiotherapy, injections) except bracing may introduce significant variability and confounding. This needs to be critically discussed as a methodological limitation.”*

We have added wording to a sentence in the limitations section of the Discussion (on page 37, lines 722-723) to acknowledge these limitations in addition to the factors already discussed.

*“Baseline Characteristics Table (Table 2): Should report p-values to confirm baseline comparability between groups.”*

We will refrain from reporting p-values for baseline comparability, as current guidance from the CONSORT statement and related literature recommends against statistical testing of baseline characteristics in randomized trials. Since randomization accounts for differences due to chance, such tests can be misleading. Instead, we have provided descriptive statistics for baseline characteristics, as per CONSORT guidelines.

*“Units of Measurement: All variables should have clearly stated units (e.g., meters, degrees, cm).”*

&

*“Consistency: Data formats (mean (SD), n (%)) should be standardized across all tables.”*

We have checked all our texts and tables and were unable to find any unclear or inconsistent reporting of the units of measurement or data formats. If there are any of these issues indeed, specific guidance (e.g. page- and line numbers, table numbers) from the reviewer would be appreciated to assist in properly addressing this issue.

*“Footnotes: Should provide expanded explanations regarding missing data.”*

We have changed the wording of the notes ^c^ and ^d^ below Table 2 (page 18, line 312-314) to explain that this data was missing due to incomplete assessments by the assessor.

*“Adjusted Analyses: Table 3 (co-interventions) should ideally include or at least discuss adjustment analyses for potential confounders.”*

Thank you for spotting this omission. We have added a sentence to the ‘Co-interventions’ section (page 21, line 371-374) to explain what was done.

*“Figures, especially the CONSORT diagram,* *should be provided at higher resolution (minimum 300 dpi) to ensure clarity.”*

The CONSORT diagram (Figure 1) was already provided at a 600 dpi resolution as per the request of the editorial staff at initial submission. We are not sure how to improve clarity any further. Please advise.

*“Figure legends should be expanded to be fully self-explanatory.”*

We have reviewed the legends and believe they already include the necessary information for interpretation without referring to the main text. However, we are happy to make further improvements and would appreciate more specific guidance on which figure(s) or aspects the reviewer feels requires clarification or additional detail.

*“**In-text references to figures should be clearer.”*

We have reviewed the manuscript and ensured that figures are clearly referenced and appropriately placed within the text to support the relevant content. We had already included explicit citations to Figure 1 on page 6 (line 125) and on page 16 (line 293) and to Figure 2 on page 19 (line 319) and 20 (lines 342 and 347). We would appreciate any specific suggestions on where the references could be made clearer or more helpful to the reader, and we are happy to revise accordingly.

*“The final analyzed sample size (n=46) was notably lower than planned, leading to an underpowered study. The risk of Type II error should be more strongly emphasized.”*

Please see our response to comment 1 of reviewer #1 which was similar in nature. Sentences were amended (on page 36) to emphasise this more clearly.

*“Line 124–149: Randomizing participants before full eligibility confirmation led to post-randomization exclusions, which can introduce selection bias.”*

Admittedly, our procedure of randomisation prior to the baseline visit/assessment was not ideal. However, we argue that this did not introduce selection bias because most of the 16 patients that were excluded after randomisation (please see the legend of Figure 1 on page 7) were screening errors and not eligible for participation in the first place. Of these 16, there were two participants who refused to be a waitlist control participant and two who withdrew for unknown reasons. The latter two may have dropped out because of disappointment with group allocation, but we don’t know. If participants assigned to a control group drop out because they were hoping to receive the intervention, the control group may end up consisting of less motivated or different-profile participants compared to the intervention group. Therefore we already added a sentence to the limitations section of the Discussion (page 36, line 718) to acknowledge that these few participants may have led to attrition bias.

*“Line 127: Neither participants nor assessors were blinded, increasing the risk of measurement bias, particularly for subjective outcomes like VAS.”*

We have rewritten the first part of the limitations section of the Discussion (page 36, line 707-710) to acknowledge this more clearly.
