# Supplementary material for: Effectiveness and user experiences of a valgus brace in patients with knee osteoarthritis: A mixed-method randomised controlled trial
Source: PLoS One. 2025 Sep 4;20(9):e0330157. doi: 10.1371/journal.pone.0330157 (PMC12410808; doi:10.1371/journal.pone.0330157)
Supplement: Attachment — Submitted filename: Response to Reviewers_R2.docx [file pone.0330157.s010.docx]

**Response to Reviewers_R2**

PONE-D-25-04724 (PLOS ONE)
Effectiveness and user experiences of a valgus brace in patients with knee osteoarthritis:

a mixed-method randomised controlled trial.

*Editorial comments* and subsequent reply and/or amendment

*“Journal Requirements: Please review your reference list to ensure that it is complete and correct. If you have cited papers that have been retracted, please include the rationale for doing so in the manuscript text, or remove these references and replace them with relevant current references. Any changes to the reference list should be mentioned in the rebuttal letter that accompanies your revised manuscript. If you need to cite a retracted article, indicate the article’s retracted status in the References list and also include a citation and full reference for the retraction notice.”*

We have reviewed the reference list and to our knowledge is complete and correct.

Compared to our first submission, we have not retracted or amended any cited papers.

*Comments Reviewer #1* and subsequent reply and/or amendment

*“Reporting and Handling of Missing Data. Although imputation methods for questionnaire subscales (e.g., WOMAC, SF-12) are described, the manuscript still does not present a clear account of missing data rates for the primary and secondary outcomes (e.g., VAS, 6-MWT) across all timepoints. Moreover, it is not explicitly stated whether all available outcome data were included in the multilevel models using full information maximum likelihood (FIML), which is standard under the missing at random (MAR) assumption. Please report the number of participants contributing data at each assessment timepoint for each outcome. Clearly state whether linear mixed models used all available repeated measures data under the MAR assumption, and confirm that no listwise deletion or case-wise exclusion was applied. If any dropout was non-random or outcome-related, briefly discuss the potential implications for bias.”*

We used all available outcome data as this is standard when using the ‘lmer’ function in R. We have added wording to the *‘Quantitative data collection and analysis’* paragraph (page 14, line 252-253) to state explicitly we have used the ‘full information maximum likelihood’ approach.

Please note that missing data rates could already be derived from the raw data presented on the second tab of the S1 File. For clarity, there we have now also highlighted all missing data points in red and added the corresponding sample sizes (*n*) per outcome and per group in a row below the raw data.

There was no non-random dropout.

*“Post Hoc Power or Precision Interpretation. The study acknowledges being underpowered relative to its initial target (n=80), yet the current version does not provide any post hoc calculation of detectable effect size or interpretive guidance based on the observed confidence intervals. This omission limits the ability to interpret the nonsignificant results for secondary outcomes and assess the likelihood of Type II error. Please include a brief post hoc calculation of the minimum detectable effect size (e.g., for VAS difference at 6 months) given the final sample size (n=23 per arm) and original assumptions. Alternatively, summarize the observed width of the 95% confidence intervals for key outcomes to contextualize the statistical precision and interpretability of the findings.”*

As explained in our previous response, we strongly feel that adding an effect size such as Cohen’s *d* does not not enhance interpretability in the context of our multilevel model analysis with repeated measures. However, to enhance interpretability of the outcomes, we have added the mean between-group differences and their 95% CIs for VAS to the explanatory texts alongside Figure 2A and 2B, and amended the texts in the ‘Interpretation’ column (F) on the third tab of the S1 File.

*Comments Reviewer #2* and subsequent reply and/or amendment

*“1. Resolution of Figures (CONSORT Diagram). Please confirm that the resolution of Figure 1 (CONSORT diagram) is at least 600 dpi and suitable for printing. If any issues with image clarity are identified, provide a higher-quality version.*

We hereby confirm that the Figure 1 resolution is 600 dpi, which can also be checked in the ‘Properties’ tab of the image (please see the print-screen below).

*“2. Description of Biomechanical Mechanism of the Brace. In the Methods section (page 8, lines 157–159), add one or two sentences briefly describing the biomechanical mechanism of the SecuTec® OA brace (e.g., how it applies a valgus force to reduce load on the medial knee compartment). This will enhance replicability and reader understanding.”*

We have added text (on page 8, line 160-163) describing this.

*“3. Reporting of Missing Data. Report the exact extent of missing data for each outcome (e.g., VAS, WOMAC, SF-12) in the main text or a supplementary table. This will increase transparency and allow readers to better assess the impact of missing data.*

Please note that this information could already be derived from the raw data presented in the second tab of the S1 File. For clarity, there we have now also highlighted all missing data points in red and added the corresponding sample sizes (*n*) per outcome and per group in a row below the raw data.

*“4. Integration of Quantitative and Qualitative Data. Add a brief paragraph in the Results or Discussion section to summarize the findings of S2 Table (triangulation of quantitative and qualitative data) and highlight how it informs result interpretation.*

*Additionally, provide a simple comparative table to confirm that the characteristics of the interview subsample (e.g., age, gender, disease severity) do not significantly differ from the main sample.”*

We have added a concluding paragraph to the Results section (page 31/32, lines 593–611) summarising the information presented in S2 Table. Additionally, we have inserted two sentences into the Discussion section (page 34, lines 662-663 and page 35, lines 681–682) to further support the interpretation of these findings.

Regarding the request for a comparative table of interview subsample characteristics, we believe this would duplicate the information already provided in Table 2. Also, we have only provided descriptive statistics for baseline characteristics and we will refrain from statistically testing baseline comparability as current guidance from the CONSORT statement and related literature recommends against statistical testing of baseline characteristics in randomized trials. Since randomization accounts for differences due to chance, such tests can be misleading.

*“5. Effect Size. Although your rationale for not reporting Cohen’s d in multilevel models is sound, please calculate and report an approximate effect size (e.g., Cohen’s d) for the primary outcome (VAS pain after 6-MWT) in the S1 File, accompanied by a brief explanation of its limitations in the context of multilevel models.”*

We have added the mean between-group differences and their 95% CIs to the explanatory texts alongside Figure 2A and 2B, and amended the texts in the ‘Interpretation’ column (F) on the third tab of the S1 File.

*“6. Units and Data Format Consistency. Review all tables and text to ensure that units (e.g., meters, degrees, centimeters) are clearly stated for all variables and that data formats (e.g., mean (SD) or n (%)) are consistent across all tables and text. Correct any inconsistencies identified.”*

As indicated in our previous ‘Response to Reviewers’, specific guidance (e.g. page- and line numbers, table numbers) from the reviewer would be appreciated to assist in properly addressing this issue. We have checked all our texts and tables once more and were unable to find any unclear or inconsistent reporting of the units of measurement or data formats.

*“7. Figure Legends. Expand the legend for Figure 2 (page 19, lines 325–349) to clearly explain the meaning of black and gray dots/lines, the range of scores (e.g., VAS, SF-12, WOMAC), and any other relevant details. This will ensure the figure is fully self-explanatory.”*

We have tweaked the legend of Figure 2 (page 19, line 336-344) in the hope this is now sufficiently self-explanatory.

*“8. Explanation of Sample Size Reduction. Although reasons for participant exclusion post-randomization are provided in Figure 1 (page 7, lines 134–149), please add a brief sentence in the Discussion section (near lines 707–710, page 36) to clarify the practical reasons for the reduced sample size (e.g., logistical constraints or COVID-19 impact). This will provide additional context for readers.”*

We have expanded and tweaked a sentence in the Discussion (page 37, line 734-737) to clarify this.

*“9. Discussion of Disease Severity (Kellgren & Lawrence Grade). The Results section (page 21, lines 376–379) notes that a higher percentage of participants with Kellgren & Lawrence Grade 3 in the intervention group underwent TKA or UKA post-study.* *Please add a brief sentence in the Discussion section to address this observation, noting its potential impact on brace effectiveness as a limitation or direction for future research.”*

We have added a sentence to the Discussion on page 38 (line 761-763) to discuss this specific observation.

*“10. Clarification of Qualitative Analysis Approach. In the Methods section (page 23, lines 271–290), add a brief sentence explaining why a deductive approach was chosen over an inductive approach for the qualitative analysis. This will help readers understand the methodological decision.”*

We have added a sentence in the ‘Qualitative data collection and analysis’ paragraph (page 15, line 275-277) to explain this.
